# Recent advances in catalytic asymmetric synthesis

**DOI:** 10.3389/fchem.2024.1398397

**Published:** 2024-05-09

**Authors:** Ashna Garg, Dominick Rendina, Hersh Bendale, Takahiko Akiyama, Iwao Ojima

**Affiliations:** ^1^ Stony Brook University, Department of Chemistry, Stony Brook, NY, United States; ^2^ Gakushuin University, Department of Chemistry, Tokyo, Japan; ^3^ Stony Brook University, Institute of Chemical Biology and Drug Discovery, Stony Brook, NY, United States

**Keywords:** asymmetric catalytic synthesis, asymmetric organocatalysis, asymmetric photocatalysis, asymmetric electrocatalysis, biocatalysis, C-H activation, flow chemistry

## Abstract

Asymmetric catalysis stands at the forefront of modern chemistry, serving as a cornerstone for the efficient creation of enantiopure chiral molecules characterized by their high selectivity. In this review, we delve into the realm of asymmetric catalytic reactions, which spans various methodologies, each contributing to the broader landscape of the enantioselective synthesis of chiral molecules. Transition metals play a central role as catalysts for a wide range of transformations with chiral ligands such as phosphines, *N*-heterocyclic carbenes (NHCs), etc., facilitating the formation of chiral C-C and C-X bonds, enabling precise control over stereochemistry. Enantioselective photocatalytic reactions leverage the power of light as a driving force for the synthesis of chiral molecules. Asymmetric electrocatalysis has emerged as a sustainable approach, being both atom-efficient and environmentally friendly, while offering a versatile toolkit for enantioselective reductions and oxidations. Biocatalysis relies on nature’s most efficient catalysts, i.e., enzymes, to provide exquisite selectivity, as well as a high tolerance for diverse functional groups under mild conditions. Thus, enzymatic optical resolution, kinetic resolution and dynamic kinetic resolution have revolutionized the production of enantiopure compounds. Enantioselective organocatalysis uses metal-free organocatalysts, consisting of modular chiral phosphorus, sulfur and nitrogen components, facilitating remarkably efficient and diverse enantioselective transformations. Additionally, unlocking traditionally unreactive C-H bonds through selective functionalization has expanded the arsenal of catalytic asymmetric synthesis, enabling the efficient and atom-economical construction of enantiopure chiral molecules. Incorporating flow chemistry into asymmetric catalysis has been transformative, as continuous flow systems provide precise control over reaction conditions, enhancing the efficiency and facilitating optimization. Researchers are increasingly adopting hybrid approaches that combine multiple strategies synergistically to tackle complex synthetic challenges. This convergence holds great promise, propelling the field of asymmetric catalysis forward and facilitating the efficient construction of complex molecules in enantiopure form. As these methodologies evolve and complement one another, they push the boundaries of what can be accomplished in catalytic asymmetric synthesis, leading to the discovery of novel, highly selective transformations which may lead to groundbreaking applications across various industries.

## 1 Introduction

One of the fundamental challenges in organic synthesis is the creation of molecules with specific chirality. The synthesis of enantiopure compounds remains a significant focus in pharmaceutical research, due to the fact that each enantiomer may well have distinct metabolic and toxicological characteristics and only specific enantiomer possesses desirable pharmacological properties, while the other enantiomer may cause undesirable side effects. Thus, the use of racemic compounds as pharmaceutical drugs may impose serious risks (Ceramella et al., 2022). The production of enantiomerically pure drugs is often time-consuming, costly, and environmentally deleterious (Meggers, 2015). The use of chiral auxiliaries or enantiomerically pure starting materials from natural sources is costly in general and inefficient. Therefore, the development of highly efficient methods and processes in catalytic asymmetric synthesis has profound significance in the pharmaceutical industry, which needs to develop efficacious and safe chiral drugs with high target specificity (Betz et al., 2023).

In 2022, a book, “Catalytic Asymmetric Synthesis, fourth Edition” edited by Akiyama and Ojima, was published, which provided a comprehensive overview of the advances in the field between 2010 to early 2020 (Akiyama and Ojima, 2022). Since the advancement in this field of research is continuous, very fast and highly robust, numerous publications emerged since early 2020. Accordingly, the purpose of this review article is to focus on the most significant advances in catalytic asymmetric synthesis in the last 5 years (2018–2023) in specific areas, i.e., organocatalysis, photocatalysis, electrochemical catalysis, biocatalytic transformations, and applications of these catalytic processes in continuous flow system.

This review also intends to showcase how different chiral catalysts can be synergistically used in addressing complex synthetic challenges (Emmanuel et al., 2023; Malakar et al., 2023). The development of these protocols has been spearheading the development of sophisticated, but cost-friendly and environmentally benign processes in catalytic asymmetric synthesis (Guo et al., 2022; Nagib, 2022; Wang et al., 2023). Judicious combination of various chiral catalysts and catalytic asymmetric processes would substantially expand chemist’s toolbox for the design and synthesis of target molecules with chiral centers bearing required absolute configurations.

Where a drug’s efficacy and safety hinges on its purity and stereochemical integrity, continuous flow processes can provide precise control over the quality of products (Tsubogo et al., 2015). There has been a substantial advancement in continuous flow processes since Kobayashi and coworkers demonstrated the potential of these systems in practical catalytic asymmetric synthesis in 2013 (Tsubogo et al., 2013). This area of research involves chemistry, chemical engineering and computer science, aiming at rendering chemical manufacturing processes more efficient, safe and eco-friendly (Plutschack et al., 2017).

Catalytic asymmetric synthesis appears to have a bright future not only for the productions of pharmaceuticals, diagnostics and materials, but also for the advancement of chemical sciences through new discoveries and innovative applications (Yamamoto and Ishihara, 2008; Reyes et al., 2022).

## 2 Asymmetric organocatalysis

### 2.1 Enamine/iminium catalysis

Enamine species are prevalent in literature as a key intermediate for a diverse array of asymmetric organocatalytic transformations. In 2000, List, Lerner, and Barbas reported their proline-catalyzed intermolecular aldol condensation with an enamine species as a key intermediate (Bahmanyar and Houk, 2001; Sakthivel et al., 2001; Mukherjee et al., 2007) and in the same year MacMillan reported a Diels–Alder cyclization catalyzed by a chiral imidazolidinone via an iminium ion intermediate (Sakthivel et al., 2001; Erkkilä et al., 2007; Mukherjee et al., 2007). Since initial discoveries in 1970s (Hajos ZGP and Parrish, 1974), proline-derived organocatalysts have been indispensable tools in asymmetric catalytic transformations with some advantages associated with their usage, e.g., reactions proceed under mild and aerobic conditions, wherein moisture is tolerated. In 2021, the Nobel Prize in Chemistry was awarded jointly to Benjamin List and David MacMillan, highlighting the importance of asymmetric enamine/iminium organocatalysis as a versatile and environmentally benign chemical process, effective for complex organic transformations in laboratories and industry (Mukergee, 2021).

**FIGURE 1:**
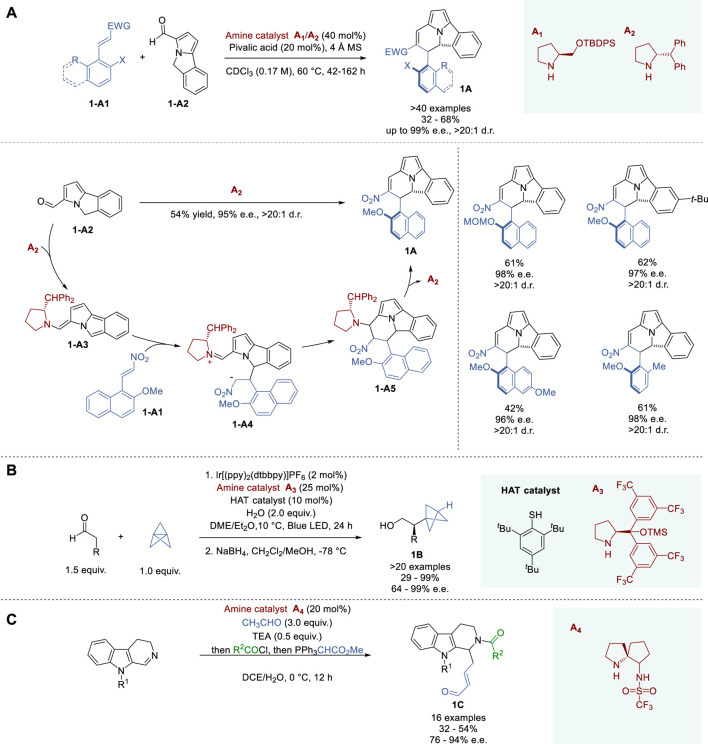
Asymmetric enamine-iminium catalysis **(A−C)**.

In 2022, Jørgensen and coworkers succeeded in synthesizing atropoisomeric cyclizine cores with a conformationally stable C (sp^2^)-C (sp^3^) stereogenic axis for the first time, via enantioselective cyclization (Bertuzzi et al., 2022). This reaction took place between 5*H*-benzo [*a*]pyrrolizine-3-carbaldehydes and nitroolefins, α,β-unsaturated ketoesters or α,β-unsaturated aldehydes, and represents the first example of highly enantioselective synthesis of cyclazine cores. For the reaction of **1-A1** with **1-A2**, a modified amine catalyst **A**_**2**_ was found to be optimal, which gave cyclizine **1A** in 54% yield and 95% e. e. As Figure 1A shows, the condensation of aldehyde **1-A2** with organocatalyst **A**_**2**_, produces enamine **1-A3**, followed by C-C bond formation with nitroolefin **1-A1** to afford **1-A4**. Then, **1-A4** cyclizes to give **1-A5**, and the subsequent elimination of organocatalyst **A**_**2**_ yields **1A**. This reaction tolerated a diverse scope of aldehydes, nitroolefins with different *O*-protecting groups, and naphthalene substitutions, giving the corresponding cyclizine products **1A** in 32–68% yields with 92–99% e. e. and 10:1∼>20:1 d. r.

In 2021, Anderson and coworkers reported a multi-catalytic strategy, incorporating organo-, photo- and hydrogen atom transfer (HAT) catalysis to synthesize α-chiral bicyclo [1.1.1]pentanes (BCPs) **1B**, which are important bioisosteres for 1,4-disubstituted arenes, alkynes, and *tert*-butyl groups (Figure 1B) (Wong et al., 2021). The three catalytic cycles operate in unison without side reactions. During the optimization of reaction conditions, it was confirmed that iridium photocatalyst, Jørgensen-Hayashi's catalyst **A**_**3**_ and HAT catalyst were essential to obtain BCPs in 29–99% yield and 64–98% e. e. This multi-catalyst process is applicable to the asymmetric synthesis of various α-chiral BCPs bearing various functional groups at the α-position. For example, α-chiral BCP aldehydes were further derived into carboxylic acids, secondary amines, secondary alcohols, and homologated to alkynes without erosion of enantiomeric purity.

In 2019, Guo and coworkers reported a bifunctional enamine catalyst in the course of the total synthesis of naucleofficine I and II (Figure 1C) (Yuan et al., 2019). A trifluoromethanesufonamide group was introduced into a spirobicyclic pyrrolidine to enhance enantioselectivity. Through the optimization of this process, catalyst **A**_**4**_ was identified as optimal, which gave the key intermediate **1C** (*R* ^2^ = *t*-Boc, R^3^ = Ac) in 51% yield with 91% e. e. This asymmetric catalysis was applied to the enantioselective synthesis of a variety of substituted 3,4-dihydro-β-carbolines and indoles in 32–54% yield and 76–94% e. e.

### 2.2 Asymmetric Brønsted acid catalysis

Chiral Brønsted acid catalysts have been playing an important role in various asymmetric transformations since early 2000s (Yamamoto and Ishihara, 2008). Chiral Brønstead acids are tunable by varying the pKa, steric environment and mode of activation to produce effective catalysts for different asymmetric transformations (Akiyama and Ojima, 2022). Among a variety of chiral Brønsted acid catalysts, chiral phosphoric acids (CPAs) have been extensively studied and developed as one of the most versatile chiral catalysts. CPA is a bifunctional catalyst that functions as a Brønsted acid, as well as a Brønsted base, to form a hydrogen bonding network, involving a nucleophile and electrophile to promote asymmetric transformations (Faisca Phillips and Pombeiro, 2023). In 2004, Akiyama and Terada reported a successful application of CPAs to enantioselective Mannich-type reactions, achieving high yields and excellent enatioselectivity (Akiyama et al., 2004; Uraguchi and Terada, 2004). Chiral Brønsted acids were also designed for asymmetric counter-anion directed catalysis (ACDC), as demonstrated by the works of List, wherein chiral imidodiphosphorimidates (IDPis) were successfully used as catalysts in a variety of asymmetric transformations (Schreyer et al., 2019).

**FIGURE 2:**
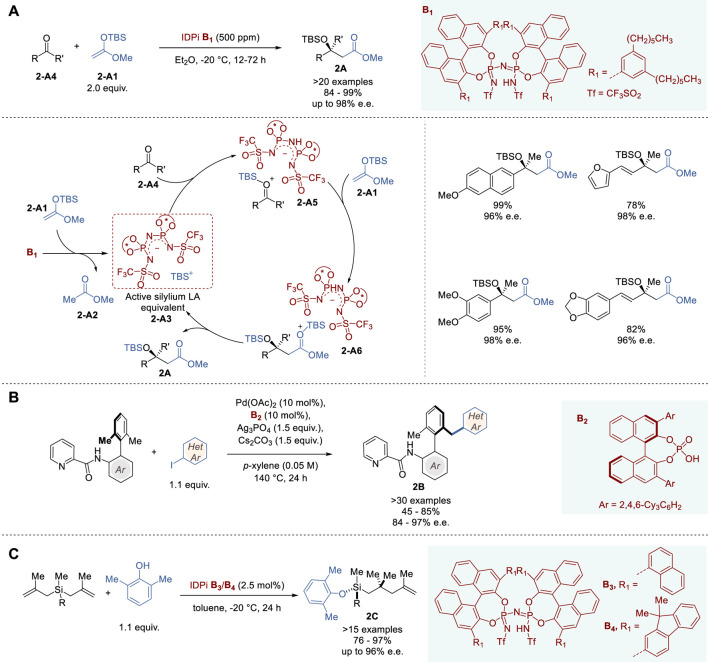
Chiral Brønsted acid catalyzed asymmetric reactions **(A−C)**.

In 2018, List and coworkers reported highly efficient silylium-based ACDC, using IDPi-based catalysts in Mukaiyama-type aldol condensation, which proceeded with a sub-ppm quantity of the catalyst in a 10 g scale reaction (Figure 2A) (Bae et al., 2018). The aldol reaction of TBS-ketene acetal **2-A1** with a variety of ketones **2-A4** using catalyst **B**_**1**_ gave the corresponding cross-aldol products **2A** in 84–99% yield and 68∼>98% e.e.

In 2023, Akiyama and coworkers reported the first example of axially chiral biaryl desymmetrization by directing group-assisted atroposelective C (sp³)-H activation using a CPA-Pd(II) catalyst (Figure 2B) (Uchikura et al., 2023). The reaction of biaryls bearing a picolinamido moiety at the 2′-position as the directing group with aryl iodides catalyzed by Pd(OAc)_2_-**B**_**2**_ with Ag_3_PO_4_ as an additive, afforded chiral biaryl products **2B** in 45–85% yield and 84–97% e.e. However, 2-methoxyiodobenzene was an exception, resulting in poor yield with enantioselectivity, due to its steric constraints. The picolinoyl directing group can be removed with zinc/HCl quantitatively to give the corresponding amine without loss of enantiopurity. The catalytic cycle, involving Pd^II^- and Pd^IV^-phosphate species, and the mechanism of enantioselection based on DFT analysis of TS-models were presented, which nicely accommodate the experimental results.

In 2022, List and coworkers reported the first organocatalytic enantioselective synthesis of tertiary silyl ethers with “central chirality” on silicon using IDPi catalysts (Figure 2C) (Zhou et al., 2022). This novel reaction involves an asymmetric desymmetrization of symmetrical bis(methallyl)silanes via carbon−carbon bond formation and silicon-hydrogen exchange reaction with phenols. The reaction of benzylbis (metallyl)silane with 2,6-dimethylphenol to give **2C** (R = Bn) was used for optimization of IDPi catalysts and found that **B**_**3**_ and **B**_**4**_ were optimal, giving **2C** with 90% e.e. and 94% e.e., respectively, in >95% yield. The reactions of substituted benzylbis(metallyl) silanes and arylbis (methallyl)silanes catalyzed by **B**_**3**_ or **B**_**4**_ gave the corresponding Si-stereogenic aryloxysilanes **2C** in 76–97% yield with 92–96% e.e. The absolute configuration of **2C** (R = 4-methylbenzyl) was determined by Fujita’s crystalline sponge method (Zigon et al., 2021). Aryloxysilanes **2C** with high enantiopurity can serve as key building blocks for a variety of applications, since **2C** can be derived to various organosilanes through functionalizations of the olefin moiety and substitutions at the aryloxy-Si bond.

### 2.3 Asymmetric Brønsted base and hydrogen-donor catalysis

In a manner similar to that of proline-derived organocatalysts, chiral Brønsted bases function as catalyst for a variety of asymmetric transformations under mild conditions. Chiral Brønsted bases typically consist of chiral amine moieties as essential components by design. This asymmetric catalysis involves rather complex hydrogen-bonding networks among the substrate, chiral catalyst and nucleophile. This multi-component process also requires a delicate balance between the basicity of the catalyst and the acidity of the nucleophile. Since a variety of chiral Brønsted bases can be rationally designed, a large number of chiral catalysts have been invented and developed (Denmark and Beutner, 2008; Ishikawa et al., 2017), which are impossible to cover comprehensively. Accordingly, only a few selected examples, including hydrogen-donor catalysis are discussed here.

**FIGURE 3:**
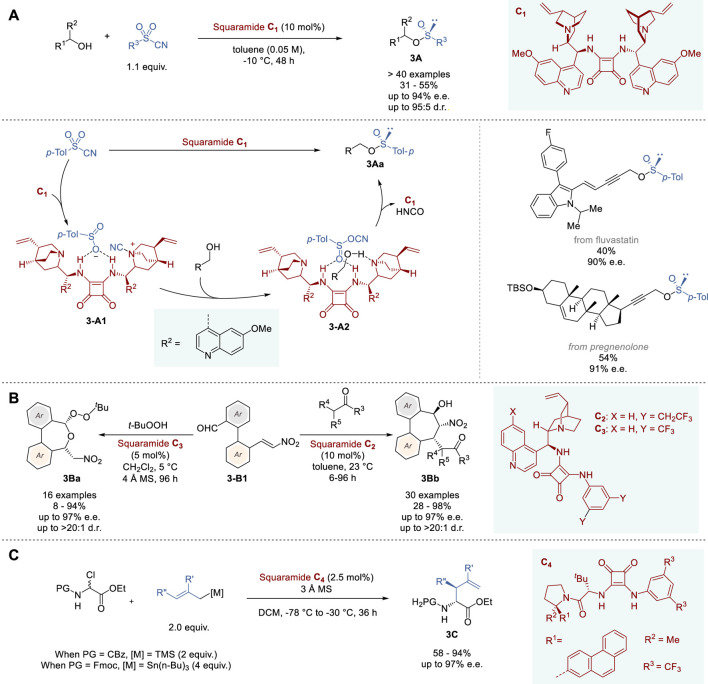
Chiral Brønsted base and hydrogen-donor catalyzed asymmetric reactions **(A−C)**.

In 2023, Yan and coworkers reported the successful transformation of achiral hexavalent sulfonyl cyanides to the corresponding tetravalent chiral organosulfinates **3A** through asymmetric deoxygenation catalyzed by novel squaramide catalyst **C_1_** (Figure 3A) (Huang et al., 2023). This unique catalysis involves the activation of tosyl cyanide through hydrogen bonding to the two NH groups of **C**_1_, which promotes the stereoselective cleavage of the S-CN bond and the migration of the CN group to the quinuclidine nitrogen (**3-A1**), followed by addition of alcohol and another migration of the CN group to the sulfinyl oxygen to form sulfinyl cyanate complexed to (**3-A2**). Then, alcohol undergoes S_N_^2^ substitution at sulfur to give chiral sulfinate **3Aa**, liberating HNCO/HOCN and catalyst C_1_. The mechanism of this catalysis was elucidated by FT-IR spectroscopy, ^18^O labeling experiment and DFT calculations. A broad range of alcohols and sulfonyl cyanides were used to demonstrate the versatility of this enantioselective catalytic process, with emphasis on the use of a variety of functionalized propargylic alcohols, which gave the corresponding sulfinates **3A** in 31–55% yield with 80–94% e. e. This process was also applied to late-stage modifications of drugs and bioactive natural products such as carbazoles, citral, β-ionone, fluvastatin, D-glucose, and pregnenolone.

In 2023, Chauhan and coworkers reported the catalytic asymmetric synthesis of 5,7-dihydrobenzo [*c,e*]oxepines (**3Ba**) bearing two stereogenic centers, as well as dibenzocycloheptanes (**3Bb**) bearing three stereogenic centers, using bifunctional quinoline-squaramide catalysts (Figure 3B) (Kotwal et al., 2023). The bifunctional catalyst was designed to bear two different reaction sites, i.e., aldehyde and nitrovinyl groups, at the two *ortho* positions of the biaryl ring system (**3-B1**). After complexation of **3-B1** to bifunctional catalyst **C**_**2**_ or **C**_**3**_, a soft nucleophile, a β-keto ester, β-diketone or amide, undergoes a domino 1,4/1,2-addition to afford dibenzocycloheptanes **3Bb**, while a hard nucleophile, t-BuOOH, proceeds with a tandem 1,2/oxa-Michael addition to give dihydrobenzooxepines **3Ba**. An optimization study initially identified bifunctional Brønsted base catalyst **C**_**2**_ as optimal for the domino 1,4/1,2 addition to give dibenzocycloheptanes in 28–98% yields with 93–97% e. e. and >20:1 d. r. Further tuning of **C**_**2**_ for tandem 1,2/oxa-Michael addition led to **C**_**3**,_ which was found to be the optimal catalyst for this process, affording various oxepines **3Ba** in 8–94% with 83–97% e. e. and 4:1∼>20:1 d.r. The relative and absolute configuration of **3Ba** and **3Bb** were determined by X-ray crystallography and DFT analysis revealed that these reactions are thermodynamically controlled processes.

In 2018, Jacobsen and coworkers reported the enantio- and diastereoselective synthesis of α-allyl amino esters **3C** through allylation of *N*-Cbz- or *N*-Fmoc- α-chloroglycinates by hydrogen-donor catalysis of squaramides (Figure 3C) (Bendelsmith et al., 2019). The reaction of ethyl *N*-Cbz-α-chloroglycinate with 2-methallyltrimelthylsilane was used to screen squaramide catalysts, which identified **C**_**4**_ as the optimal catalyst. A variety of allylsilanes and allylstannanes were employed as allylating agents, wherein ethyl *N*-Fmoc-α-chloroglycinate was used for the reaction with allylstannanes, to give the corresponding *N*-Cbz-/*N*-Fmoc-α-allylglycinates **3C** in 58–94% yield with 90–97% e. e. and >10:1 d. r. A mechanistic study, including kinetics experiments and DFT calculations, suggests a concerted S_N_2 process with dynamic kinetic resolution.

### 2.4 Asymmetric *N*-heterocyclic carbene catalysis

*N*-Heterocyclic carbenes (NHC) can be generated *in situ* through the deprotonation of the corresponding various “azolium” salts (Flanigan et al., 2015). Since Ukai’s pioneering work on an NHC generated from a thiazolium salt in benzoin condensation 80 years ago (Ukai et al., 1943) and Breslow’s work on the mechanism in 1958 (Breslow, 1958), chiral NHCs appeared already in 1960–1970s and have been contributing to the advancement of catalytic asymmetric synthesis (Flanigan et al., 2015). The field of chiral NHC catalysis appears to be still expanding its boundaries in asymmetric organocatalysis.

**FIGURE 4:**
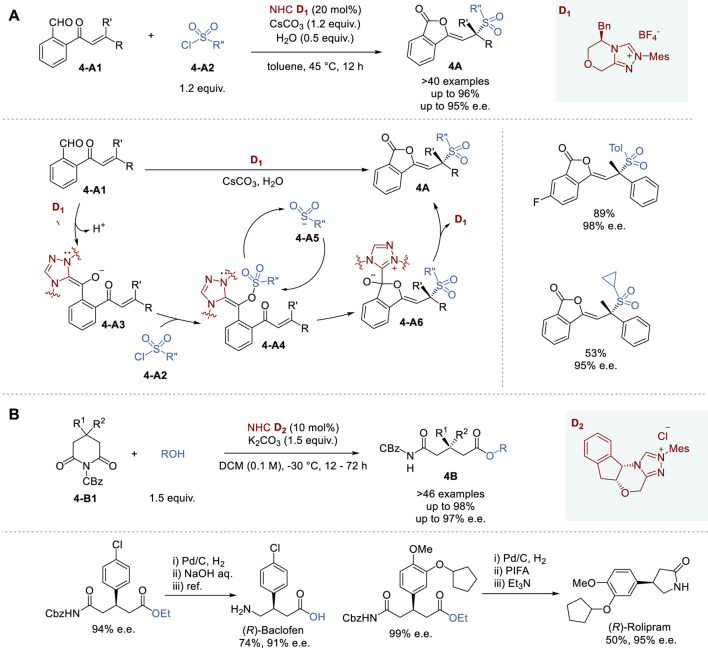
Chiral NHC catalyzed asymmetric synthesis **(A,B)**.

In 2022, Chi and coworkers reported the enantioselective sulfonylation of 2-(substituted acryloyl)benzaldehydes catalyzed by chiral NHC species generated from triazolium pre-catalyst **D**_**1**_ to give the corresponding sulfonylethylideneisobenzofuranones **4A** in 20–95% yield and 87–98% e.e. (Figure 4A) (Deng et al., 2022). Since chiral sulfones are unique functional groups found in pharmaceuticals and natural products, this process may provide an efficient access to biologically active sulfone-containing compounds. In this NHC catalysis, sulfonyl chloride functions as both an oxidant and a nucleophile via its reduced form. The chiral NHC species generated from pre-catalyst **D**_**1**_ reacts with the aldehyde moiety to initiate the activation of sulfonyl chloride, generating a sulfinate species that undergoes Michael addition to a remote enone moiety stereoselectively, triggering the cascade cyclization to give the product **4A** and regenerate the chiral NHC catalyst. Mechanistic studies, including DFT calculations, suggest the involvement of an unprecedented Breslow intermediate and a novel mode of oxidation.

Although successful chiral NHC catalyzed asymmetric reactions typically involve aldehydes, enals and esters as substrates, the reactions involving amides are still challenging. Nevertheless, in 2022, Huang and coworkers reported a successful protocol for the enantioselective desymmetrization of 4-substituted and 4,4-disubstituted *N*-Cbz-glutarimides (**4-B1**) with alcohols under mild conditions in the presence of triazolium pre-catalyst **D**_**2**_ (Figure 4B) (Hu et al., 2022). This process includes the enantioselective cleavage of the imide C-N bond by chiral NHC species generated from **D**_**2**_, followed by ester formation with an alcohol to give the corresponding glutarate-*N*-Cbz-amide **4B**. A structurally diverse 3-substituted and 3,3-disubstituted glutarate-*N*-Cbz-amides **4B** were synthesized by this process in 40–97% yield and 46–98% e. e. Furthermore, this process was successfully applied to the synthesis of the key intermediates of (*R*)-Baclofen (skeletal muscular relaxant) in 74% yield and 91% e. e., and (*R*)-Rolipram (antidepressant) in 50% yield and 95% e. e.

### 2.5 Hypervalent iodine catalysts in asymmetric synthesis

**FIGURE 5:**
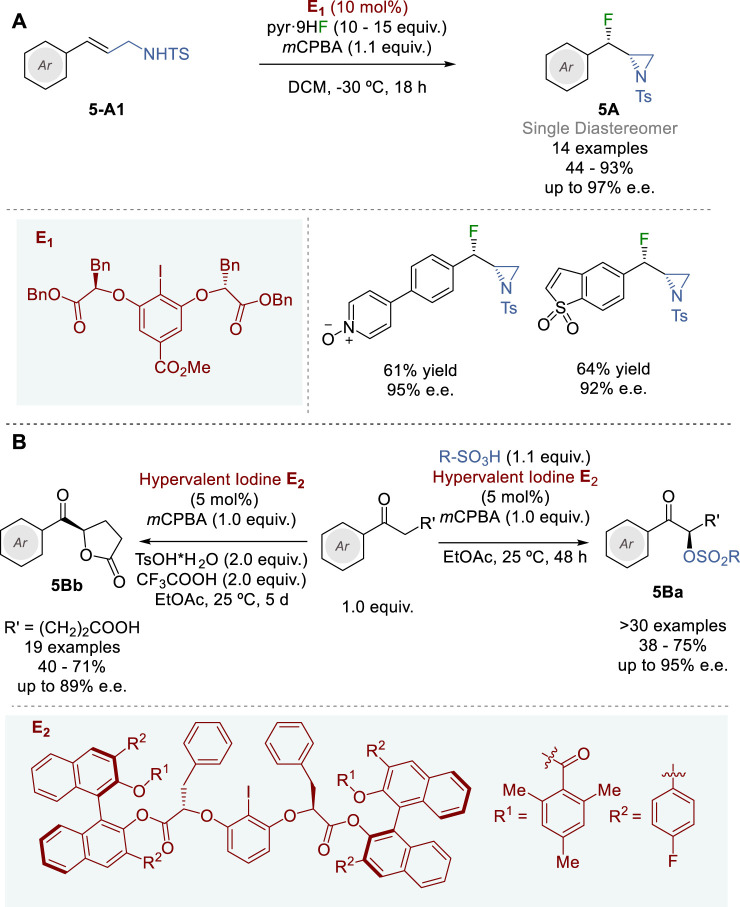
Hypervalent iodine catalyzed asymmetric transformations **(A,B)**.

In 2018, Jacobsen and coworkers reported the synthesis of *syn*-β-fluoroaziridines **5A** through diastereo- and enantioselective fluorination-aziridination of *N*-tosyl-3-arylprop-2-enylamines (**5-A1**) with *m*CPBA (stoichiometric oxidant) and HF-pyridine (nucleophilic fluoride source) promoted by chiral aryl iodide catalyst **E**_**1**_, which generates hypervalent iodine species (Figure 5A) (Mennie et al., 2018). A variety of allylamines **5-A1** bearing substituted aryl and fused hetero-bicyclic aryl groups were employed as substrates in this reaction to give the corresponding fluoroaziridines **5A** in 44–93% yield and 61–97% e. e. with perfect diastereoselectivity.

Since the catalyst-controlled diastereoselectivity in this process is extremely high, the fluorination of chiral 1-substituted *N*-tosyl-3-arylprop-2-enylamines afforded the corresponding 1,3-difluoro-2-amines bearing three contiguous stereocenters with very high diastereoselectivity (>20:1). This process was also successfully applied to the fluoroamination of *N*-tosyl-3-nitrophenylpent-4-ylamine to give the corresponding *anti*-β-fluoropyrrolidine in 82% yield with 86% e. e. and >20:1 d. r. Furthermore, variants of this process were applied to allyl benzyl ether, allyl carbamate and allyl acetate to give the corresponding 1,2-oxyfluorinated products in 64–77% yield and 92–94% e.e.

Inspired by the pioneering works of Fujita (Fujita et al., 2010) and Ishihara (Uyanik et al., 2010) on chiral hypervalent iodine catalysts, and the work of List and Corić on confined Brønsted acids with BINOL-derived CPAs (C et al., 2012), Zhang and coworkers developed novel two-layer chiral aryl iodide catalysts (**E**_**2**_ and variants) in 2023 (Figure 5B) (Zhang et al., 2023a). The salient feature of this catalyst design includes the highly confined 2-iodoresorcinol core, which is linked to two sterically demanding BINOL derivatives by two chiral α-benzylglycolate arms. The first layer, consisting of 2-iodoresorcinol core linked to two chiral α-benzylglycolate moiety serves as the chiral environment for hypervalent iodine catalysis and the second layer, consisting of the finely tuned BINOL-derivatives drastically restricts the flexibility of the first layer, locking the highly demanding chiral environment at the iodine site. Through tuning of various BINOL derivatives the catalyst **E**_**2**_ was identified as optimal and used in the asymmetric α-oxysulfonylation of alkyl aryl ketones to give the corresponding α-sulfonyloxyketones **5Ba** in 38–75% yield and 73–95% e.e. Catalyst **E**_**2**_ was also applied to the oxidative enantioselective lactonization of various 5-oxo-5-arylpentanoic acids to afford various arenecarbonyl- and 5-heteroarenecarbonyl-γ-butyrolactones **5Bb** in 40–71% yield and 71–89% e. e.

## 3 Asymmetric visible-light photoredox catalysis

Visible-light photoredox catalysis represents a fundamental departure from traditional methods of activating chemical reactions. Visible light, which is abundant and non-destructive, is an ideal energy source for these processes. The process involves a catalyst that absorbs visible light, triggering an electron transition from HOMO to LUMO (Li et al., 2020). Then, the excited catalyst species thus formed can undergo intersystem crossing to reach a triplet excited state (T_1_), which is more stable than the singlet excited state (S_1_). This approach offers several appealing and complementary advantages over conventional ground-state catalysis. It facilitates the generation of reactive radical species under mild reaction conditions, thereby enabling unique transformations that can rapidly generate molecular complexity and the late-stage functionalization of intricate molecules (Albini and Fagnoni, 2004; Prier et al., 2013; Schultz and Yoon, 2014). Establishing enantioselective photoredox-catalyzed processes is challenging in organic synthesis, given that intermediate radicals are highly reactive and reactions involving these species have low energy barriers (Shaw et al., 2016). This eventually leads to side reactions that are not enantioselective. Even with these problems, new ways of controlling the stereochemistry in photoredox processes have emerged in the last 10 years, facilitating the efficient synthesis of chiral molecules (Yoon, 2016; Saha, 2020; Yao et al., 2022).

A key to the successful asymmetric photoredox catalysis is the selection of an appropriate catalyst. A typical photoredox catalyst is a transition metal complex or an organic dye that facilitates electron transfer processes, generating radical intermediates (Prier et al., 2013). This activation has enabled countless synthetic transformations. Merging photocatalysis with either a transition metal or an organocatalyst leads to reactions that neither catalyst could achieve alone (Prier et al., 2013). Transition metal complexes, such as those based on noble metals, e.g., platinum, rhodium, ruthenium and iridium, have demonstrated remarkable efficiency in a wide range of transformations (Lee and Lo, 2022). However, this section will focus on the potential of economically favorable transition metals such as copper and nickel, which mediate organic transformations, achieving high enantioselectivity.

In addition to examples that demonstrate how photoredox catalysis has been effectively combined with traditional metal catalysis, the synergism between photocatalysis and organocatalysis will be highlighted in this section.

### 3.1 Asymmetric metallaphotoredox catalysis

**FIGURE 6:**
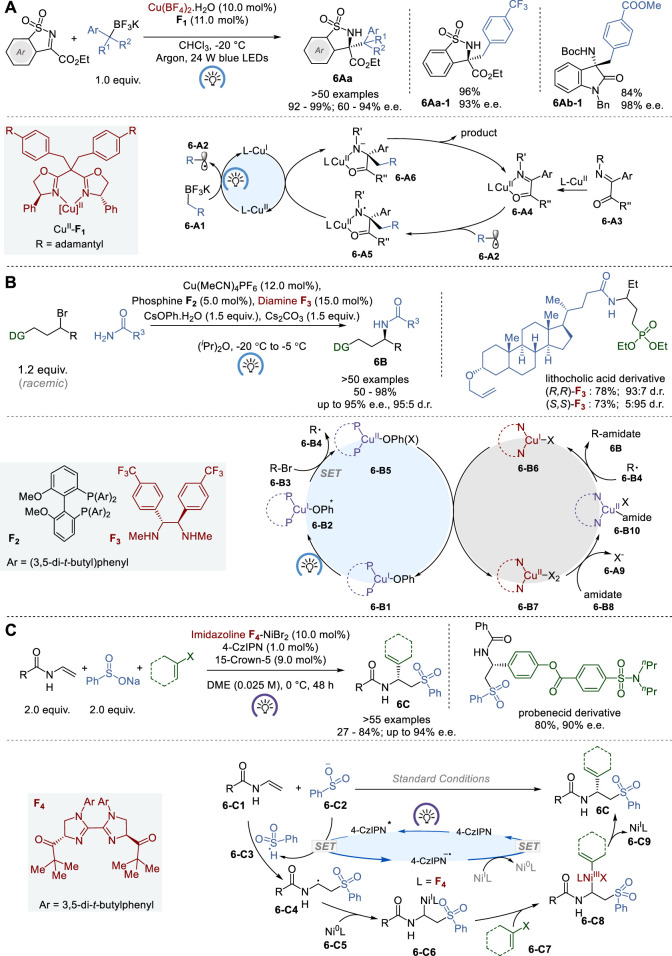
Metallaphotoredox asymmetric catalysis **(A−C)**.

In 2018, Gong and coworkers reported an enantioselective light-induced alkylation of *N*-sulfonylimines with benzyl trifluoroborates catalyzed by copper (II)-bisoxazoline complexes (Cu^II^-BOX), which was generated *in situ* from Cu(BF_4_)_2_ and BOX ligands (Figure 6A) (Li et al., 2018). The Cu^II^-BOX complexes (e.g., Cu^II^-**F**_**1**_) act as chiral photoredox bifunctional catalysts in this process to give various chiral *N*-sulfonylamines **6Aa** with tetrasubstituted carbon stereocenters in 92–99% yield and 60–94% e. e. This reaction was also successfully applied to the asymmetric alkylation of isatin-derived ketimines to afford the corresponding 3-*N*-*t*-Boc-amino-3-alkyloxindoles **6Ab** in 69–84% yield and 96–98% e.e.

Transition-metal catalysis in the asymmetric synthesis of chiral alkylamines, involving alkyl electrophiles and nitrogen nucleophiles, is gaining traction as a promising synthetic approach to higher-order amines (Trowbridge et al., 2020). However, the progress has been rather limited except for asymmetric allylic aminations (Grange et al., 2016). In 2021, Fu and coworkers reported a novel enantioconvergent amidation of unactivated racemic alkyl electrophiles using a photoinduced copper catalyst system (Figure 6B) (Chen et al., 2021). The process relies on three distinct ligands, i.e., bisphosphine **F**_**2**_, phenoxide and chiral diamine **F**_**3**_, and these ligands form two distinct catalysts, i.e., (i) a copper/bisphosphine/phenoxide complex that acts as a photocatalyst **6-B1** and (ii) a chiral copper/diamine complex **6-B6** that catalyzes enantioselective C-N bond formation. This novel process gives a variety of chiral secondary amides **6B** with high enantiopurity up to 95% e. e. through coupling of primary amides with unactivated racemic electrophiles, expanding enantioselective *N*-substitution by alkyl electrophiles beyond activated electrophiles. Traditional S_N_2 reactions have limited utility when dealing with less reactive electrophiles, particularly those that are sterically hindered. Also, it is noteworthy that conventional substitution methods seldom provide the ability to manipulate the stereochemistry at the carbon atom of the newly formed C-N bond when using a readily accessible racemic electrophile. Thus, this process marks a significant breakthrough in synthetic methodology.

In 2023, Nevado and coworkers reported a novel asymmetric three-component carbosulfonylation of *N*-vinyl amides and *N*-vinyl carbamates (**6-C1**) with sodium benzenesulfinate and aryl/vinyl halides, using a dual nickel/photoredox catalyst system, to give the corresponding chiral 2-aryl/alkenyl-2-amidoethylsulfones **6C** in 27–84% yield and 76–94% e.e. (Figure 6C) (Du et al., 2023). This process allows for the concurrent formation of C−C and C−S bonds across the alkene’s π-system with enantiocontrol. With a broad substrate scope and high functional group tolerance, this process has been proven effective for the synthesis of pharmacologically pertinent 2-aryl-2-amidoethylsulfones. The mechanism of this dual catalysis includes a chiral biimidazoline (BiIM) **F**_**4**_-NiBr_2_ catalyst cycle for asymmetric cross-coupling and 4-CzIPN for photoredox catalyst cycle. BiIM **F**_**4**_ was selected over several ligands screened for this process as the best chiral ligand so far. The proposed mechanism involves the formation of a secondary alkyl radical **6-C4**, which is captured by Ni^0^L (L = **F**_**4**_) **6-C5** to generate Ni^I^ complex **6-C6**. Then, aryl/alkenyl halide **6-C7** undergoes oxidative addition to generate Ni^III^ complex **6-C8**, which subsequently undergoes reductive elimination to give the product **6C** and Ni^I^-L species, **6-C9**. Finally, **6-C9** is reduced to Ni^1^-L by photoredox catalysis to regenerate Ni^0^-L catalyst **6-C5**. The proposed Ni^0^/Ni^I^/Ni^III^ catalytic cycle is supported by various control experiments, including radical scavengers, cross-over and control experiments.

### 3.2 Asymmetric photoredox organocatalysis

**FIGURE 7:**
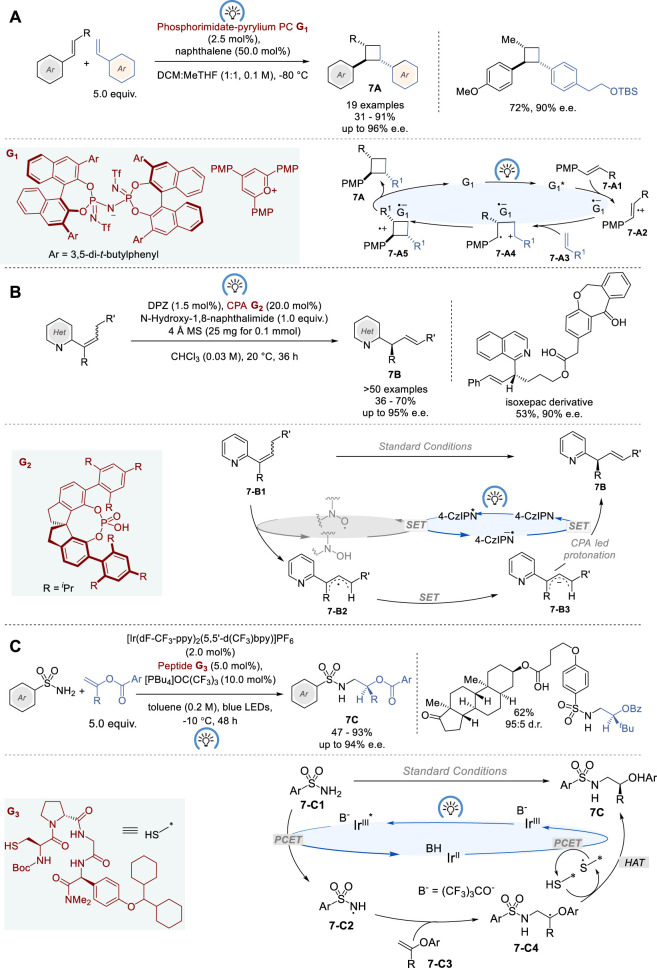
Photoredox organocatalysis for asymmetric synthesis **(A−C)**.

The advancement of asymmetric photoredox catalysis has gained utmost recognition in recent years (Shaw et al., 2016). Asymmetric organocatalysis, including enamine, iminium-ion, Brønsted acid/base, and *N*-heterocyclic carbene catalysis, has been used to induce chirality transfer in photocatalytic reactions (Yoon, 2016; Zou et al., 2018). In general, asymmetric photoredox catalysis needs a second activation mode to facilitate asymmetric induction, due to the absence of general methods to control the stereochemistry of radical ion species (Nicewicz and MacMillan, 2008; Proctor et al., 2018; Huan et al., 2021; Sherbrook et al., 2021). However, in 2023, List and coworkers reported a single-catalyst solution for enantioselective [2 + 2] cross-cycloaddition of styrenes using a chiral organic salt **G**_**1**_ with confined imidodiphosphorimidate (IDPi) counteranions as the catalyst (Figure 7A) (Das et al., 2023). A broad range of mono/di-substituted styrenes with different electronic properties were employed in the reaction with *trans*-anethole derivatives to give the corresponding chiral cyclobutanes **7A** in 31–91% yield and 76–96% e.e., wherein various functionalities, including alcohol, silyl ether, aldehyde, ester, and terminal olefin, were well tolerated. Mechanistic investigations suggest that the first step in the catalytic cycle, generating cation radical intermediate complexed to **G**_**1**_ (**7-A4**), determines the enantioselectivity, and the second step, forming the cyclobutane cation radical complexed to **G**_**1**_ (**7-A5**), determines the diastereoselectivity, resulting in thermodynamically and kinetically favored C-C bond formation between two benzylic sites in a *trans*-configuration. Finally, single electron transfer from the counter anion radical of **G**_**1**_ * to the cyclobutane cation radical gives product **7A**.

The catalytic asymmetric olefin isomerization to create a chiral tertiary carbon stereocenter is a highly atom economical process to produce chiral alkenes that are versatile intermediates in organic synthesis. Thus, this process has attracted considerable interest over the past couple of decades, wherein asymmetric hydride transfer catalysis (Tani et al., 1982; Tani et al., 1984; Tanaka and Fu, 2001) and bio-inspired enantioselective proton-transfer catalysis (Wu et al., 2011; Lee and Deng, 2012; Morack et al., 2021) are found to be effective. However, thermodynamic advantage of the product over the starting alkene is naturally required for this process to proceed, and thus the substrate type is structurally limited. Accordingly, it is necessary to overcome thermodynamic barrier to expand this process.

In 2023, Jiang and coworkers provided a partial solution to this challenge by applying a dual catalyst system, comprising visible light photosensitizer 5,6-bis(5-methoxythiophen-2-yl)pyrazine-2,3-dicarbonitrile (DPZ) as photoredox catalyst, chiral phosphoric acid (CPA) **G**_**2**_ and *N*-hydroxyimide as the hydrogen atom transfer (HAT) catalyst (Figure 7B) (Liu et al., 2023). A wide range of conjugated α-substituted γ-arylalkenylazaarenes (*E/Z*-mixture) were successfully isomerized by this dual catalyst system to give the corresponding chiral γ-arylallylazaarenes **7B** bearing α-tertiary carbon stereocenters in 36–70% yield and 66–95% e. e. This process was also applied to the synthesis of α-deuterio-γ-arylallylazaarenes using D_2_O as the deuterium source with 95% deuterium incorporation and 90–94% e. e, although 21–32% of the starting material was recovered. The proposed mechanism suggests that this transformation is triggered by the reductive quenching of DPZ*, which allows the generation of the crucial imide-*N*-oxyl radical (PINO) from *N*-hydroxynaphthalimide through single-electron oxidation. Following the abstraction of γ-H from **7-B1** by PINO, the resulting neutral radical intermediate **7-B2** is reduced by DPZ−•, to generate anion **7-B3**. Then, anion **7-B3** undergoes a highly α-regioselective and enantioselective protonation to give product **7B** catalyzed by CPA **G**_**2**_. The proposed mechanism also explains the reason why this process does not achieve full conversion, due to the regioselectivity issue at the protonation of **7-B3**.

In 2023, Knowles and coworkers reported enantioselective radical-based hydroamination of enol esters with sulfonamides, using a triple catalyst system consisting of an Ir photocatalyst, a Brønsted base, and a tetrapeptide thiol catalyst for asymmetric HAT (Figure 7C) (Hejna et al., 2023). This reaction provides easy access to a variety of chiral β-amino alcohol derivatives, i.e., α-substituted β-arylsulfonamidoethyl benzoates **7C**, in 47–93% yield and 73–94% e. e. In this catalysis, the absolute configuration of the product **7C** is determined by enatioselective HAT from the chiral peptide thiol catalyst to a prochiral C*-*centered radical **7-C4**. The optimization of tetrapeptide thiol HAT catalyst identified Boc-Cys-^D^Pro-Acpc-^X^Phg-NMe_2_ (X = *p*-Cy_2_CH-O) **G**_**3**_ to be optimal. Computational and experimental mechanistic study on this triple catalysis revealed that hydrogen-bonding, π−π stacking, and London dispersion interactions play key roles for substrate recognition and induction of chirality by chiral tetrapeptide HAT catalyst in this process.

## 4 Asymmetric electrochemical catalysis

The coupling of electrochemistry with chiral transition metal catalysis or organocatalysis has been recognized as a powerful strategy for asymmetric electrochemical catalysis, leveraging precise control over redox processes to drive enantioselective reactions.

Electrosynthesis uses electricity instead of chemical reagents to transform organic molecules, which involves oxidation at the anode and reduction at the cathode in a conductive medium and is executed at either constant current or potential. Electrosynthesis can be carried out in a cell with or without a membrane, separating the anodic and cathodic spaces. The electrochemical transformations can be conducted directly at the electrode or indirectly using a redox mediator in the solution, and the latter can enhance efficiency and selectivity under milder conditions. Thus, catalytic asymmetric electrosynthesis can be realized by the combination of a chiral catalysts and a redox mediator (Yan et al., 2017; Shah and Ngai, 2022). This approach is more economically feasible and versatile than the use of specialized chiral electrodes, electrolytes, solvents or pre-modified chiral substrates (Arnaboldi et al., 2018). Moreover, electrochemical synthesis can be effectively combined with various asymmetric catalyst systems, such as transition metal-catalysts and organocatalysts, as well as photochemical and bioelectrochemical asymmetric synthesis (Kärkäs, 2018; Kingston et al., 2019; Zhu et al., 2021).

### 4.1 Asymmetric electrochemical organocatalysis

Shono oxidation is an effective electrochemical process for α-functionalization of amines with a variety of nucleophiles to capture the *in situ* formed iminium ion species (Jones and Banks, 2014; Zhu et al., 2021). This oxidation process is significant in organic synthesis to construct complex nitrogen-containing molecules.

**FIGURE 8:**
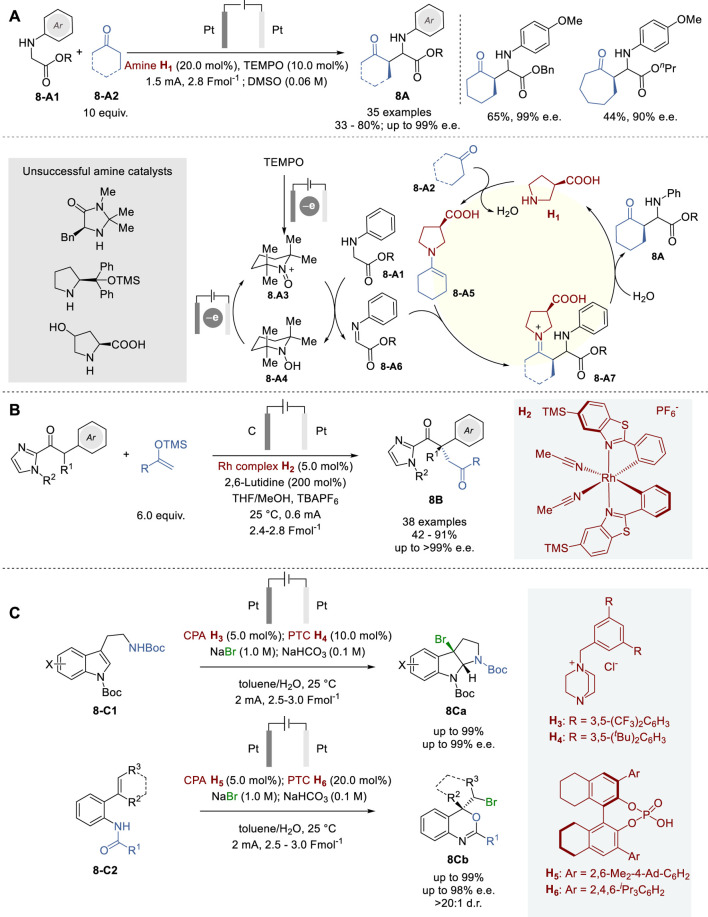
Eelectrochemical organocatalysis for asymmetric synthesis **(A−C)**.

In 2021, Mei and coworkers reported an electrochemical asymmetric coupling of secondary acyclic amines **8-A1** with ketones **8-A2** via Shono-type oxidation for the formation of amino acid derivatives **8A** (Figure 8A) (Wang et al., 2021). In this process, an *N*-oxyl radical TEMPO was used as the redox mediator for anodic oxidation, which enables selective oxidation of *N*-arylglycinate substrates, but not α-substituted products **8A** by exploiting a slight potential difference between the two. The use of TEMPO provided better functional group tolerance, as compared to the use of stoichiometric additives such as metals, electrolytes, and oxidants. For the substrate scope of this process, a variety of *N*-anisylglycinate derivatives, as well as cyclohexanone, cycloheptanone, tetrahydro-4-pyranone and tetrahydro-4-thiopyranone were employed to give the corresponding *N*-aryl-α-cycloalkylglycinates in 33–80% yield with diastereoselectivity up to >99:1 d.r. and enantioselectivity up to 99% e.e.

The proposed catalytic cycle commences with the oxidation of TEMPO at the anode, forming oxoammonium species **8-A3**, which reacts with a glycine ester to produce TEMPO-H (**8-A4**) and iminoester **8-A6**. On the other hand, enamine **8-A5**, formed from ketone **8-A2** and amine catalyst **H**_**1**_, reacts with **8-A6** to generate iminium **8-A7**. Hydrolysis of **8-A7** gives the product **8A** and regenerates catalyst **H**_**1**_. In the meantime, protons at the cathode are reduced to hydrogen, preventing them from interfering with the catalytic cycle.

Although combining organic electrosynthesis with asymmetric catalysis is a highly promising strategy, there are challenging issues that impede its progress. For instance, the radical or ion intermediates, generated via oxidation or reduction electrochemically, need to be connected to an enantioselective catalyst cycle. Otherwise, it will lead to non-catalyzed side reactions, impacting yield and enantioselectivity. Furthermore, certain chiral catalysts may have limited compatibility with an electrochemical cell conditions.

In 2019, Meggers and coworkers introduced a simplified strategy to overcome these challenges, which is termed “electricity-driven chiral Lewis acid catalysis”, using a cationic chiral-at-metal rhodium catalyst **H**_**2**_ for the oxidative cross-coupling of 2-acyl imidazoles with silyl enol ethers, producing 1,4-dicarbonyl compounds **8B** (Figure 8B) (Huang et al., 2019). A broad substrate scope was shown with 38 examples to give the corresponding 1,4-dicarbonyl compounds **8B**, including those bearing all-carbon quaternary stereocenters, in 42–91% yields and 90∼>99% e. e. A novel chiral cationic Rh complex H_2_ acts as a Lewis acid to activate 2-acyl imidazole substrate toward anodic oxidation by raising the HOMO on Rh enolate formation, which enables mild redox conditions to avoid undesirable side reactions, leading to high levels of chemo- and enantioselectivities, as well as high functional group tolerance.

In 2023, Sun and coworkers reported a combinatory catalysis approach for asymmetric bromocyclizations of tryptamines (**8-C1**) and 2-benzamidostyrenes (**8-C2**) to give the corresponding products **8Ca** and **8Cb** with excellent enantiopurity, using a phase transfer catalyst (PTC), CPA, and electrochemical oxidation (Figure 8C) (Tan et al., 2023). Bromine anion from NaBr is converted to Br_2_ by anodic oxidation in aqueous phase, which is captured by PTC catalyst (**H**_**3**_ or **H**_**4**_) to form PTC-Br_2_ species. Then, the PTC-Br_2_ species interacts with lipophilic CPA catalyst (**H**_**5**_ or **H**_**6**_) to form an organic-soluble CPA/PTC-Br_2_ ion pair, which is transferred to the organic layer to trigger the asymmetric bromination of **8-C1** or **8-C2** without interference from the electrochemical system in the aqueous layer. After bromination, organic product remains in the organic layer, but HBr generated and the ionic PTC catalyst move back to the aqueous layer, where HBr is neutralized by NaHCO_3_ and converted to H_2_ (cathode) and Br_2_ (anode) which is captured by PTC, forming PTC-Br_2_ to complete the catalytic cycle. This process is applicable to a good number of tryptamines (**8-C1**) and 2-benzamidostyrenes (**8-C2**) to give the corresponding 3-bromotetrahydropyrroloindoles **8Ca** (90–99% yield and 76–99% e.e.) and 4-bromomethy-2-phenylbenzooxazines **8Cb** (93–99% yield 89–98% e.e.). This process was also successfully applied to tryptophol derivatives and tryptophan derivatives, as well as the synthesis of a key intermediate (99.5% yield and 90% e.e.) in the total synthesis of (−)-chimonanthine and (−)-hodgkinsine.

### 4.2 Asymmetric metallaelectrocatalysis

Asymmetric metallaelectrocatalysis is another successful combination of transition-metal catalyzed asymmetric synthesis with electrochemistry, which has proven to be highly effective, as evidenced by its growing use in organic synthesis (Chakraborty et al., 2021). Although the use of heterogenous metal reductants such as Mn^0^ and Zn^0^ is a common practice to generate and maintain the active catalyst species in transition-metal catalyzed processes, it is associated with some major issues, such as variability in activity due to different sources, batches, storage conditions, stirring conditions, production of excess waste, etc. Thus, more environmentally friendly and efficient alternative to heterogenous metal reductants is desirable.

**FIGURE 9:**
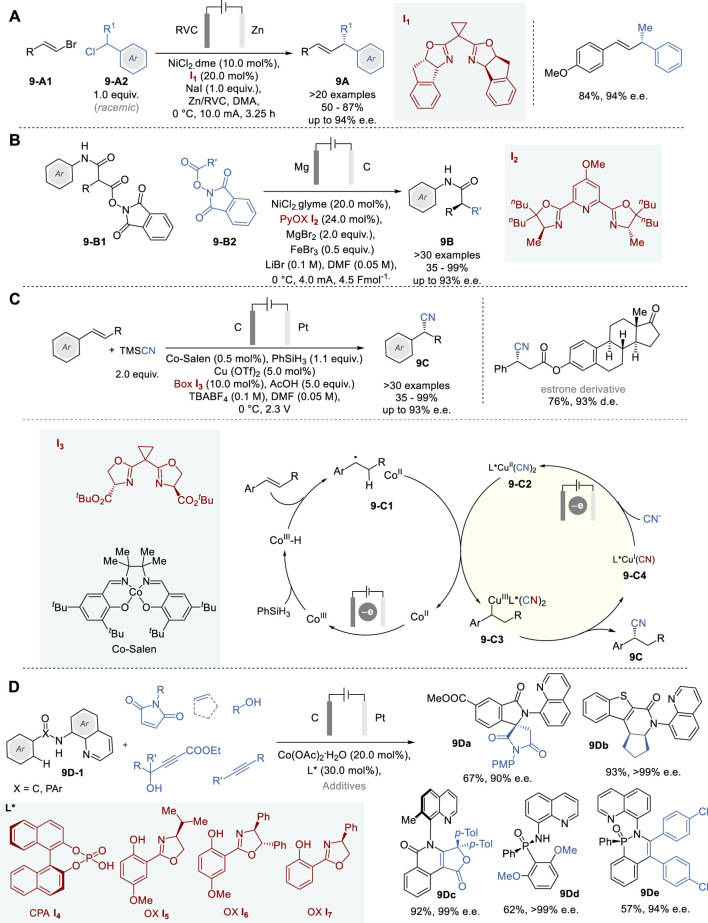
Metallaelectrocatalytic asymmetric synthesis **(A−D)**.

In 2019, DeLano and Reisman reported a novel nickel complex-catalyzed enantioselective electroreductive cross-coupling of alkenyl bromides **9-A1** and racemic α-substituted benzyl chlorides **9-A2** to give the corresponding α-substituted allylbenzenes **9A** with good structural diversity in 50–87% yield and 80–94% e. e. (Figure 9A) (DeLano and Reisman, 2019). This Ni catalysis includes NiCl_2_⋅dme complex with an indanyl-substituted bis(oxazoline) ligand **I**_**1**_ as the precursor of the active chiral Ni^0^ catalyst, which is generated by effective electrolysis using a cell equipped with an RVC cathode, a sacrificial Zn anode, and NaBr as an effective additive, as well as an electrolyte.

In 2023, Baran and coworkers reported enantioselective doubly decarboxylative C (sp^3^) −C (sp^3^) cross-coupling of the redox-active esters (i.e., *N*-hydroxyphthalimide esters) of malonic acid half amide (**9-B1**) and those of primary carboxylic acids (**9-B2**) by means of Ni-electrocatalysis to give the corresponding amides with α-alkylated stereocenters **9B** (Figure 9B) (Gao et al., 2023). This novel process includes a NiCl_2_⋅glyme complex with a new PyBox-based chiral ligand **I**_**2**_ as the chiral metal catalyst, MgBr_2_, FeBr_3_, and LiBr as electrolytes, Mg anode and RVC cathode. A broad scope of redox-active substrates was used to give the corresponding α-alkylated amides **9B** in 35–99% yield with up to 80–93% e.e. Furthermore, notable advantages of this process were demonstrated by the highly efficient short synthesis of medicinally relevant key intermediates in the total synthesis of natural products through simplification of synthetic pathways, e.g., from 10 to 15 steps to 3–5 steps and elimination of numerous functional group manipulations.

Chiral nitriles are versatile motifs and often serve as key intermediates in the synthesis of pharmaceuticals and bioactive compounds (Fleming et al., 2010). While asymmetric transformations of C=O and C=N bonds into cyanohydrins and α-aminonitriles are well known, the asymmetric hydrocyanation of alkenes is still a challenging reaction. In 2020, Lin and coworkers reported an efficient enantioselective hydrocyanation of conjugated alkenes by means of dual electrocatalysis without stoichiometric oxidants, involving Co-catalyzed HAT and Cu-catalyzed radical cyanation, to give the corresponding α-cyanoalkylarenes (**9C**) and α-cyanoalkylalkenes/alkynes in 32–95% yield and 76–95% e. e. (Figure 9C) (Song et al., 2020). In this process, dual catalytic cycles comprise a Co^III^-Salen catalyst for HAT with PhSiH_3_ and chiral bisoxazoline-Cu(OTf)_2_ complex (**I**_**3**_-Cu) for enantioselective cyanation with trimethylsilyl cyanide (TMSCN). In the Co catalyst cycle, H radical is generated from Co^III^-Salen and hydrosilane to form Co^III^–H species via HAT, which reacts with the alkene substrate to form a carbon-centered radical **9-C1**. On the other hand, bisoxazoline-Cu(OTf)_2_ complex is converted to bisoxazoline-Cu(CN)_2_ complex by reacting with cyanide anion generated from TMSCN in the Cu catalyst cycle. Then, radical **9-C1** is captured by L*Cu^II^(CN)_2_ (L* = bisoxazoline **I**_**3**_) (**9-C2**) to generate chiral Cu^III^ species **9-C3**, followed by reductive elimination to form the cyanation product **9C** and releases L*Cu^I^(CN) catalyst **9-C4**, which is converted to **9-C2** via single electron oxidation to complete the Cu catalyst cycle.

In 2023, Ackermann and coworkers reported a novel enantioselective electrochemical Co-catalyzed dehydrogenative aryl C-H activation reaction and its applications to the enantioselective C-H/N-H annulations of carboxylic amides, affording various nitrogen heterocycles bearing point and axial chirality, as well as enantioselective desymmetrization of phosphinic amides by dehydrogenative C-H activation, yielding various P-stereogenic compounds with high enaniopurity (Figure 9D) (von Münchow et al., 2023). This work demonstrated the versatility of electro-oxidative cobalt catalysis for the synthesis of a range of complex molecules, including spirolactams (e.g., **9Da**), dihydroisoquinolinones (e.g., **9Db**), atropoisomeric furoisoquinolinones (e.g., **9Dc**), and phosphinic amides (e.g., **9Dd** and **9De**). This process shows a broad functional group compatibility and uses readily available chiral ligands **I**_**4**_∼**I**_**7**_ directly accessible from BINOL or amino alcohols, which achieved high enantioselectivity to give those products with up to 99% e.e. Also, this process is scalable to decagram-scale without any loss of efficiency and enantioselectivity. Since this process generates molecular hydrogen as the sole by-product, it has a high potential to serve as a widely useful electro-oxidative transformation with excellent sustainability.

### 4.3 Asymmetric photoelectrochemical catalysis

Molecular photoelectrocatalysis has been under rapid development, allowing access to a broad range of redox potentials, enabling oxidative transformations with mild electrode potentials (Barham and König, 2020; Wu et al., 2022). Asymmetric photoelectrochemical catalysis (PEAC) combines photoredox catalysis with asymmetric electrocatalysis to facilitate enantioselective reactions without the need for external chemical oxidants.

**FIGURE 10:**
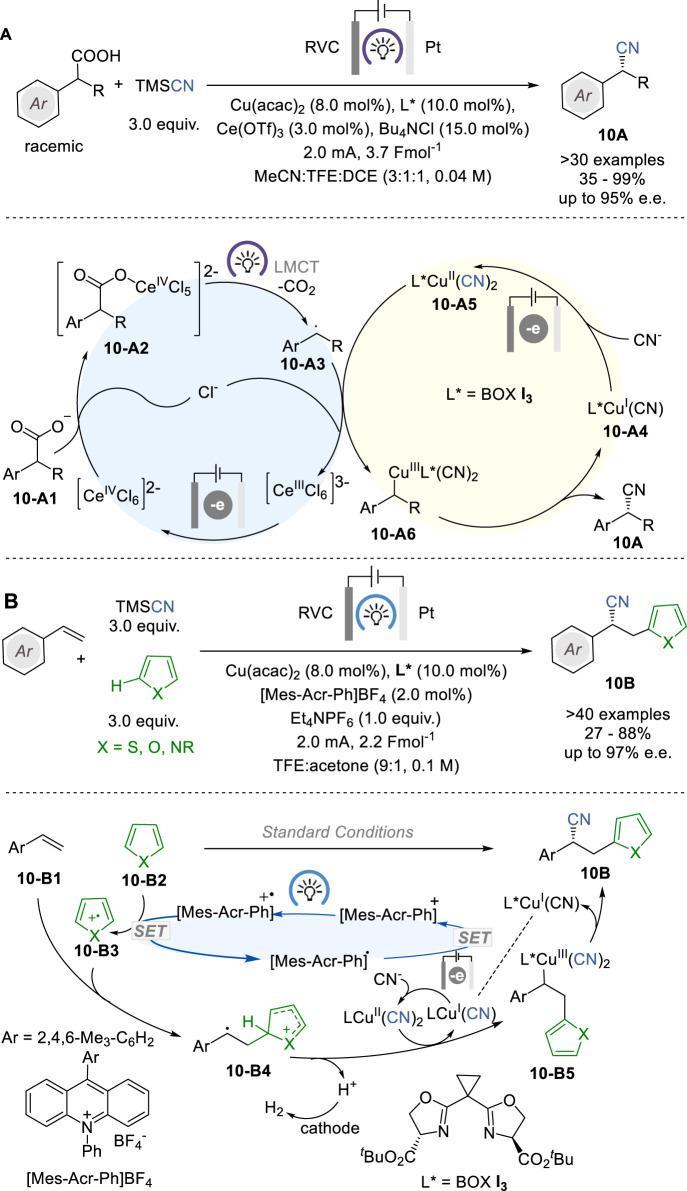
Photoelectrocatalysis for asymmetric cyanations **(A,B)**.

In 2022, Xu and coworkers reported a photoelectrocatalytic reaction that enables direct and enantioselective decarboxylative cyanation of racemic α-alkylarylacetic acids (**10-A1**) with TMSCN to give the corresponding enantioenriched α-cyanoalkylarenes (**10A**) by means of Ce/Cu relay catalysis with a Ce salt for catalytic decarboxylation and a chiral Cu complex for stereoselective C−CN formation (Figure 10A) (Lai et al., 2022). The reactions were conducted in an undivided cell equipped with a RVC anode and a Pt plate cathode and the reaction mixture was illuminated with 395 nm LEDs during electrolysis, using Ce(OTf)_3_ and Cu(acac)_2_ as pre-catalysts and chiral bisoxazoline (BOX) ligand (see **I**_**3**_ in Figure 9C) to give a variety of nitriles **10A** in 35–99% yield and 66–95% e.e.

The proposed mechanism indicates that Ce^III^(OTf)_3_ is converted to Ce^III^Cl_6_^3−^ which is oxidized to Ce^IV^Cl_6_^2−^ at the anode in the presence of chloride ion. The coordination of the carboxylate, followed by photoinduced ligand to metal charge transfer (LMCT) to regenerate the Ce^III^ species and produce a benzylic radical **10-A3** through decarboxylation. This radical **10-A3** then reacts with L*Cu^II^(CN)_2_ (**10-A4**) to produce Ar(R)CH-Cu^III^ complex (**10-A5**), which undergoes reductive elimination to give the chiral nitrile product **10A** and releases L*Cu^I^(CN) (**10-A3**). Then, **10-A3** is either anodically oxidized back to the Cu^II^ complex **10-A4** to complete the Cu catalyst cycle.

In 2023, Lai and Xu reported a photoelectrocatalytic protocol for the enantioselective heteroarylcyanation of styrene congeners with unactivated heteroarenes and TMSCN through C−H functionalization, which simultaneously introduces a heteroaryl group and a cyano group across the alkene moiety to give the corresponding α-cyano-β-heteroarylethylarenes **10B** (Figure 10B) (Lai and Xu, 2023). This process employs acridinium salt [Mes-Acr-Ph]BF_4_ as photoredox catalyst, Cu(CN)_2_ with a bisoxazoline ligand BOX **I**_**3**_ as chiral electrocatalyst, 456 nm LEDs, and TMSCN as the CN source. This novel enantioselective heteroarylcyanation reaction is scalable to gram-scale synthesis of various α-cyano-β-heteroarylethylarenes **10B** in 27–88% yield and 72–97% e. e. The chiral Cu catalyst cycle includes radical cation **10-B4** and chiral Cu^III^ species **10-B5** as key intermediates.

## 5 Asymmetric biocatalysis

By leveraging the inherent selectivity and efficiency of enzymes and other biological catalysts, biocatalytic asymmetric reactions offer a powerful tool for sustainable and efficient chemical synthesis (Hauer, 2020). In recent years, significant advances have been made in the evolution of biocatalysis (Bornscheuer et al., 2012; Bell et al., 2021). The exploration of biocatalysts underwent a transformative transition from the extraction of compounds from natural sources to the sophisticated approach of gene mining, facilitated by bioinformatics (Yi et al., 2021).

Enzymes are highly efficient as they possess a directing group, which controls selectivity, and a catalytic domain in one molecule. Moreover, an enzyme can be combined with other enzymes in a single process, augmenting their adaptability and effectiveness. Broadly, three distinct approaches have been pursued to enhance enantioselective reactions: (i) the generation of whole-cell biocatalysts by crafting designer organisms, (ii) the refinement of existing enzymes with inherent enantioselectivity for a specific process, and (iii) the evolution of novel enantioselective biocatalysts, starting from non-selective wild-type enzymes (Jaeger and Eggert, 2004).

### 5.1 Asymmetric enzymatic biocatalysis

**FIGURE 11:**
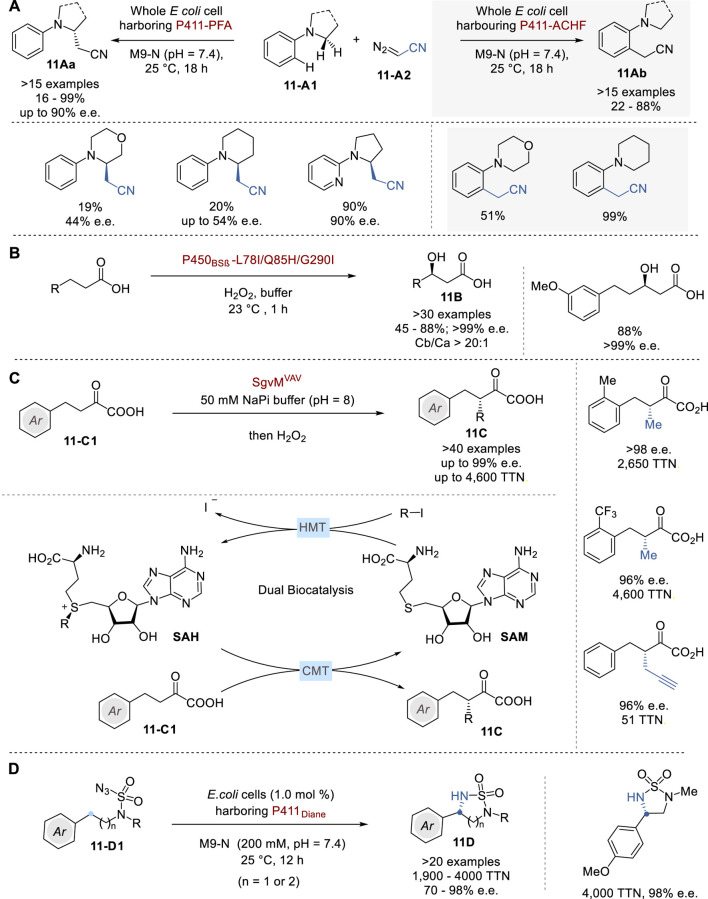
Asymmetric enzymatic biocatalysis **(A−D)**.

In 2023, Arnold and coworkers reported the selective α-cyanocarbene insertion into α-aminoalkyl C (sp^3^)-H bonds of *N,N*-dialkylaniline congeners **11-A1** via cytochrome P450 enzymes from *Bacillus megaterium* through minimal protein modifications to give enantioenriched α-cyanomethylamines **11Aa** in 16–99% yield with up to 90% e. e. (Figure 11A) (Zhang et al., 2023b). Following comprehensive crystallographic structural analysis, P411-PFA and P411-ACHF were selected as two distinct cyanomethylases. Fluoroalkylase P411-PFA effectively introduces a cyanomethyl group into the α-aminoalkyl C (sp^3^)-H bond of **11-A1** with notable chemo-, regio-, and enantioselectivity. In sharp contrast, P411-ACHF catalyzes an alkylation of the *ortho*-C (sp^2^)-H bonds of **11-A1**. This approach represents a significant advancement in the field of biocatalysis, demonstrating the enzyme’s ability to perform highly selective and efficient chemical transformations that are challenging to achieve through chemical catalysis.

The selective catalytic hydroxylation of aliphatic C (sp^3^)-H bonds in the absence of a directing group poses a significant challenge for synthetic chemists (Zhang et al., 2022). In 2022, Wang and coworkers reported an oxy functionalization of inactive C-H bonds in several aliphatic carboxylic acids by using directed evolution of P450_BSβ_ hydroxylase. This process exhibited excellent selectivity in terms of chemo-, regio-, and enantioselectivity (>30 examples, Cβ/Cα >20: 1, >99% e. e.) (Figure 11B). The X-ray crystal structure analysis of the engineered variant P450_BSβ_-L78I/Q85H/G290I, in conjunction with palmitic acid, provided a comprehensive rationale for the experimentally discerned regio- and enantioselectivity. Furthermore, the structural data revealed that a diminished catalytic pocket volume is responsible for the augmented reactivity with smaller substrates (Ma et al., 2023).

Addition of a methyl group to medicinally active molecules often greatly enhances their biological activity, a notable phenomenon commonly referred to as the “magic methyl effect” (Barreiro et al., 2011; Schönherr and Cernak, 2013). The incorporation of the smallest alkyl group into the α-position of carbonyl compounds with high enantioselectivity presents a considerable challenge for synthetic chemists. In 2023, Yang and coworkers reported a comprehensive biocatalytic framework comprising an engineered *S*-adenosylmethionine-dependent carbon methyltransferase (CMT) and a remarkably efficient halogen methyltransferase (HMT) to facilitate asymmetric β-alkylation of α-ketoalkanoic acids **11-C1** to give β-alkyl-α-ketoalkanoic acids **11C** Figure 11C) (Ju et al., 2023). CMT SgvM^VAV^ was successfully designed and implemented as a versatile biocatalyst for the enantioselective methylation, ethylation, allylation, and propargylation of a diverse array of α-keto acids, achieving total turnover numbers (TTNs) of up to 4,600 with high enantioselectivity as high as 99% e.e. The detailed analysis of crystal structures of the engineered biocatalyst unveiled two key catalytic elements, i.e., a Lewis acidic Zn site for substrate enolization and an adjacent chemical co-factor, *S*-adenosylmethionine (SAM), for stereoselective methyl transfer. Furthermore, HMTs from *Pseudomonas* bacteria, especially those from *P*. *aeruginosa*, were examined, which showed high efficiency in asymmetric methylation, highlighting the excellent potential of this biocatalytic approach (with HMT turnover up to 7,700 and SAH turnover up to 1,000). β-Alkyl-α-ketoalkanoic acids **11A** thus obtained were further transformed to various α-alkyl carboxylic acids, β-alkyl-α-hydroxy acids, and β-alkyl-α-amino acids.

In the realm of naturally occurring enzymatic C–H functionalization processes, the cytochrome P450-catalyzed C (sp^3^)–H hydroxylation stands out for its remarkable stereocontrol. Recently, various research groups have put together their efforts on reengineering these enzymes and other heme proteins and employing them to catalyze synthetically valuable reactions previously unknown to nature (Key et al., 2016; Dydio et al., 2017). Along this line, Arnold and coworkers reported a universal cytochrome P450-derived biocatalysts tailored for the enantioselective amination of primary, secondary, and tertiary C (sp^3^)–H bonds of azidosulfonylamines **11-D1** to give the chiral cyclic sulfonylimides **11D** (Figure 11D) (Yang et al., 2019). The sulfonylimides **11D** can be deprotected through treatment with 1,3-propanediamine to afford the corresponding chiral 1,2- and 1,3-diamines.

Several cytochromes, including cytochromes P450, cytochromes P411, cytochromes C, and globins, were introduced into intact *Escherichia coli* cells of which P411_Dianel_ (P411 variant lacking the FAD domain) displayed at least ten times higher activity compared to the other heme proteins, providing a TTN of 450. Under the optimized conditions, a variety of chiral cyclic sulfonylimides were obtained with TTN between 1,900–4,000 with enantioselectivity up to 98% e.e. Introduction of beneficial mutations I327P, Y263W and Q437F led to a ten-fold improvement in activity and enhanced the enantioselectivity. This best variant under optimized conditions afforded a C (sp^3^)–H amination product **11D** with 72,000 TTN and 99.9% e.e.

### 5.2 Asymmetric photoredox enzymatic biocatalysis

Nature is the best example to showcase how enzymes like fatty acid photodecarboxylase, DNA photolyase, etc., use light to facilitate biologically essential transformations (Schmermund et al., 2019). Asymmetric photoredox enzymatic biocatalysis has recently emerged as an innovative and highly promising synthetic methodology (Schmermund et al., 2019; Emmanuel et al., 2023). Merging biocatalysis with photocatalysis enables selective, light-driven transformations that offer novel reactivity, high selectivity, and better yields under environmentally benign conditions (Emmanuel et al., 2023).

**FIGURE 12:**
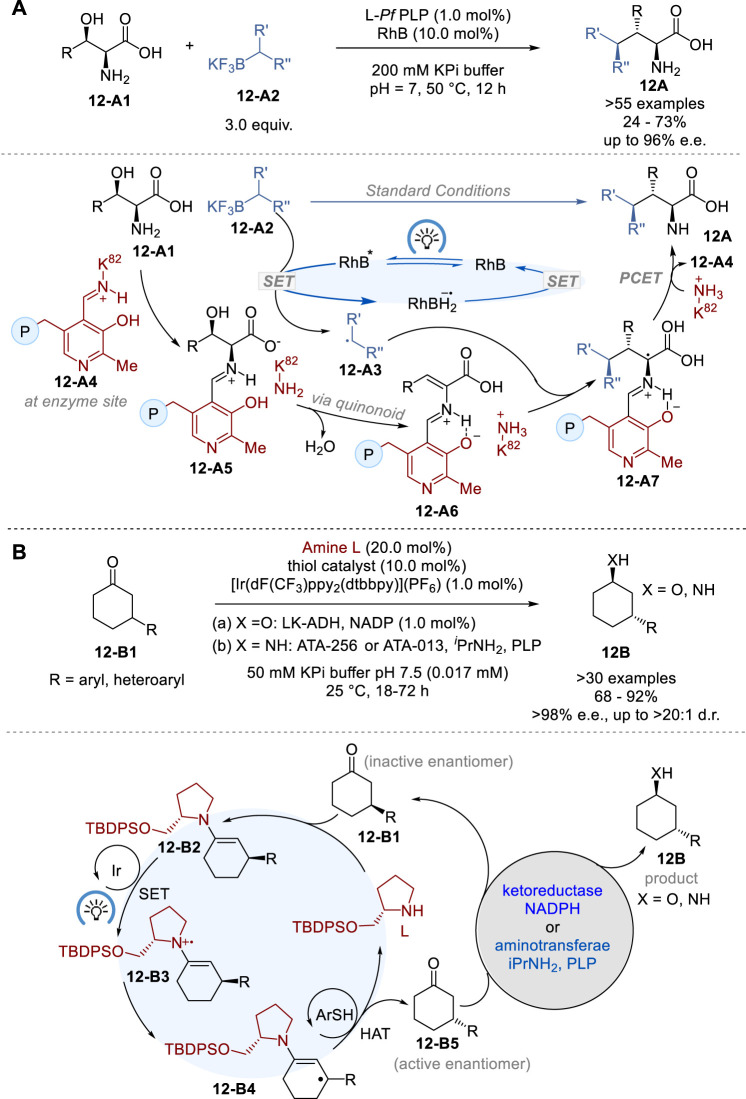
Asymmetric photoredox enzymatic biocatalysis **(A,B)**.

The conventional methods for synthesizing non-canonical amino acids (ncAAs) includes the laborious process of installation and subsequent removal of protecting groups for amine and carboxylic acid moieties (Najera and Sansano, 2007). In 2023, Yang and coworkers reported a novel approach to the diastereo- and enantioselective synthesis of ncAAs by merging photoredox catalysis with pyridoxal 5′-phosphate (PLP) biocatalysis (Figure 12A) (Cheng et al., 2023). This synergistic photoredox-pyridoxal radical biocatalysis made it possible to synthesize a variety of ncAAs in 24–73% yield with 12–96% e.e. and 10:1∼>20:1 d.r. without using any protecting groups. This dual catalysis involves the generation of carbon-centered radical **12-A3** from achiral or racemic alkylborate **12-A2** by photoredox catalyst rhodamine B (RhB) in the photoredox catalyst cycle, while free β-hydroxy-α-amino acid **12-A1** reacts with the pyridoxal-aldimine of Lys88 **12-A4** through amine exchange to form the corresponding pyridoxal-aldimine of the amino acid **12-A5**, which is dehydrated via quinonoid intermediate to form pyridoxal-aldimine of aminoacrylic acid **12-A6** in the *Pf*PLP^β^ enzyme biocatalyst cycle. Then, the radical **12-A3** is captured by **12-A6** to form the stereo-defined pyridoxal-aldimine of α-alkylated α-amino acid **12-A7**, which undergoes amine exchange with Lys88 of the enzyme to give the α-alkylated α-amino acid product **12A** and regenerate **12-A4** to complete the biocatalyst cycle.

Stereo-convergent processes offer a streamlined and simplified approach to synthetic routes, but they are often limited by the restricted range of dynamically inducible stereocenters that can be easily epimerized. In 2020, MacMillan, Hyster and their coworkers reported the successful combination of photoredox catalysis, organocatalysis and biocatalysis for the stereoconvergent synthesis of 3-substituted cyclohexanol (X = O) and cycloxylamines (X = NH) (**12B**) from 3-substituted cyclohexanones (**12-B1**) in 68–92% yield with >98% e.e., and 4:1∼>20:1 d.r. via enzymatic dynamic kinetic resolution (DKR) of the substrates bearing traditionally static, unreactive stereocenters (Figure 12B) (DeHovitz et al., 2020). This protocol employs [Ir (dF(CF_3_)ppy_)2_ (dtbbpy)]^+^ PF_6_^−^ as the photocatalyst, racemic 2-(*tert*-butyldiphenylsilyloxymethyl)pyrrolidine as the amine organocatalyst, and 4-methoxythiolphenol as the hydrogen atom transfer (HAT) catalyst for efficient racemization. A ketoreductase *Lactobacillus kefir* alcohol dehydrogenase (LK-ADH) was used for the DKR and reduction to 3-substituted cyclohexanol (**12B**, X = O), while aminotransferases, ATA-256 and ATA-013, were employed for DKR and amination to 3-substituted cyclohexylamine (**12B**, X = NH).

## 6 Asymmetric catalysis in continuous flow system

Continuous flow reactions and processes have been continuously developed, which encompass a diverse chemical transformations. The advancement in the engineering of continuous flow reactors and precise control of reaction conditions will lead to more productive and energy-efficient systems. While continuous flow processes are not always a replacement for batch processes, there are many advantages that flow chemistry can offer, including safe handling of gaseous reagents, high-pressure reactions, better mixing and heat transfer for very rapid exothermic reactions, and full-automation for higher efficiency (Plutschack et al., 2017). Flow chemistry has attracted much attention from chemists and engineers both in academia and industry, since the continuous flow systems can produce a large quantity of fine chemicals and commodity chemicals through continuous operation. As the research on continuous flow reactions has progressed (Ingham et al., 2015), naturally the applications of the continuous flow system to catalytic asymmetric transformations have attracted substantial interest among synthetic chemistry communities in the last decade, and significant advances have been made (Pastre et al., 2013).

### 6.1 Enantioselective C–C bond-formation through 1,4-addition reactions in continuous flow system

**FIGURE 13:**
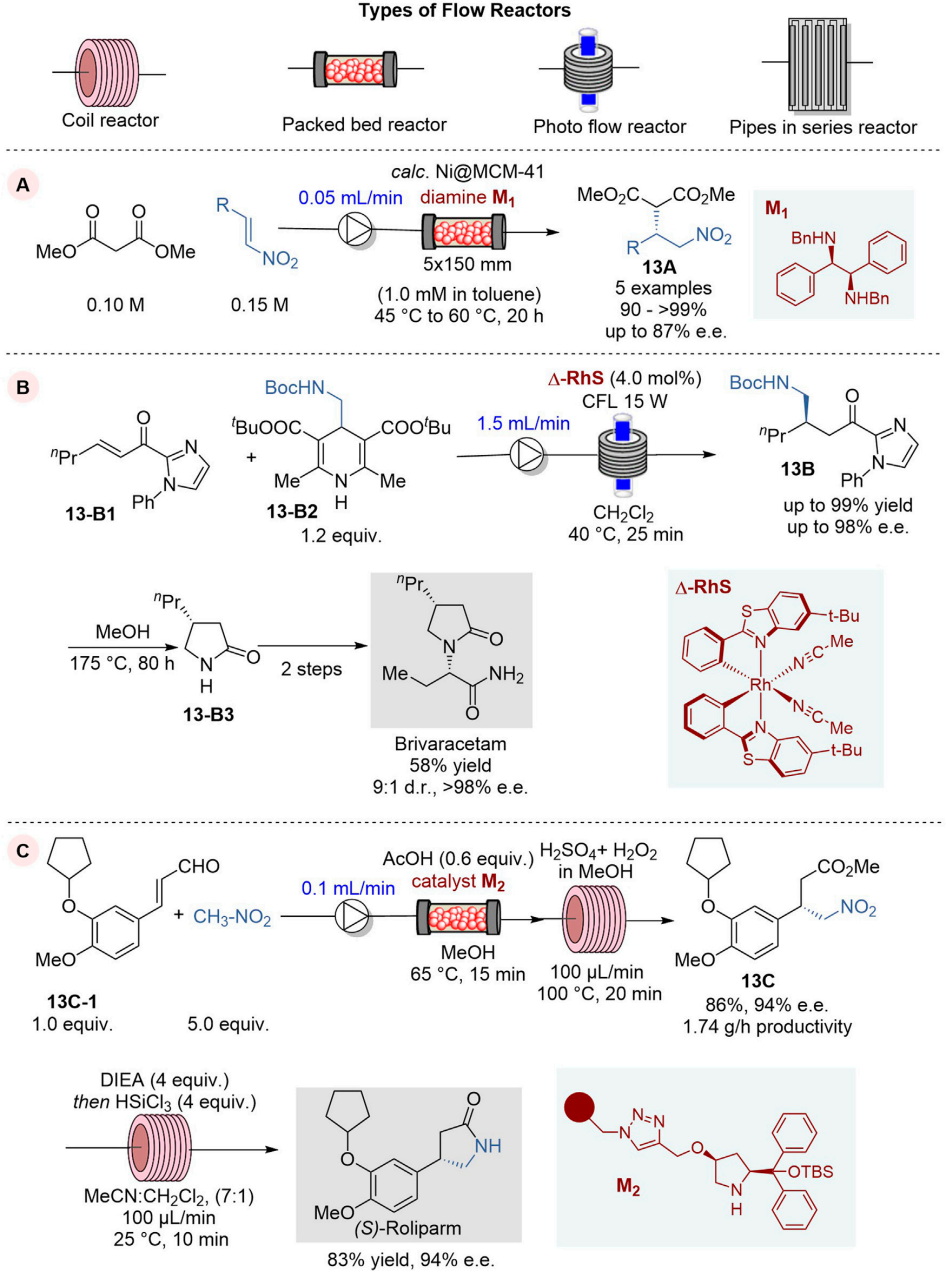
Enantioselective 1,4-addition reactions in continuous flow system **(A−C)**.

In 2023, Kobayashi and coworkers reported an enantioselective 1,4-addition of malonates to nitroolefins in a continuous flow system, using a mesoporous silica loaded with Ni salts as pre-catalyst, followed by co-feeding of chiral diamine ligand **M**_**1**_ to induce enantioselectivity to give the corresponding 1,4-addition product **13A** (Figure 13A) (Ishitani et al., 2023). This continuous flow process used a column reactor loaded with calcinated Ni pre-catalyst *calc.* Ni@MCM-41 with co-feeding of chiral ligand **M**_**1**_ and run at 45–60°C for 20 h to give 1,4-addition product **13A** in 90∼>99% yield and 73–87% e.e. (5 examples). It was found that the enantiopurity of **13A** does not change with extended operation so far up to 90 h with turnover frequency (TOF) of 3.1 h^-1^ based on Ni and a turnover number (TON) of 257, and the leaching of Ni was 1.6% after 89 h of operation.

The use of Brivaracetam as an antiepileptic drug for the treatment of drug-resistant epilepsy was approved in Europe and US in 2016 (Markham, 2016). Accordingly, the development of a practical synthetic process for Brivaracetam has been in demand and attracting considerable interest. In 2023, de Assis and coworkers reported the first application of asymmetric photocatalysis in continuous flow for the enantioselective synthesis of Brivaracetam (Figure 13B) (Franco et al., 2023) The key step in this synthesis is the enantioselective photochemical Giese 1,4-addition of BocNHCH_2_ radical, generated from Hantzsch ester **13-B2**, to *n*-hex-2-enoylimidazole **13-B1**, promoted by visible-light and chiral bifunctional Rh photocatalyst **Δ-RhS**, to give the key intermediate **13B** (de Assis et al., 2018). The key parameters for this photocatalysis were investigated and optimized in batch reactions to afford the key intermediate **13B** with 99% yield and 96% e. e. The reaction conditions were further tuned for the continuous flow process and the reaction with a flow rate of 1.0 mL/min and 4% catalytic loading of photocatalyst **Δ-RhS** at 40°C afforded **13B** in 95% yield and 95% e.e. When a blue LED lamp was substituted by a CFL lamp for better photon emission stability, the reaction gave **13B** in 94% yield and 94% e.e. in just 75 min, while the reaction in a batch reactor required 24 h to achieve a comparable result. The key intermediate **13B** was converted to another key intermediate lactam **13-B3**, followed by *N*-alkylation with (*R*)-2-bromobutanoic acid and subsequent amidation to give Brivaracetam in 58% conversion yield with 98% e. e. and 9:1 d.r.

(*S*)-Rolipram, a selective inhibitor of cAMP-specific phosphodiesterase PDE4, is an anti-inflammatory/antidepressant agent, which is often employed as a racemic mixture in biological studies. However, the pharmaceutical activity of its enantiomers was found to be divergent in many cases and thus its enantioselective synthesis needs to be developed.

In 2022, Kappe, Ötvös and their coworkers reported an efficient and green asymmetric synthesis of (*S*)-Rolipram by means of a telescoped enantioselective conjugate addition-oxidative esterification sequence followed by metal-free nitro reduction and lactamization process under continuous flow conditions (Figure 13C) (Nagy et al., 2022). The asymmetric 1,4-addition of nitromethane to cinnamaldehyde derivative **13-C1** was effectively promoted by a diphenylproline-based organocatalst on a cross-linked polystyrene-support **M**_**2**_ in an Omnifit glass column reactor at 65°C at the flow rate of 0.075 mL/min to give the corresponding chiral γ-nitro aldehyde. This aldehyde was directly delivered to a column reactor for oxidative esterification with persulfuric acid and methanol at 100°C to afford the key intermediate, γ-nitro ester **13C**, in 84% yield and 94% e. e. in a 3-h operation. In this process, hazardous and explosive persulfuric acid is safely generated in a highly controlled manner by mixing concentrated sulfuric acid and hydrogen peroxide, which is fed to the reactor with ease in a flow system. The nitro reduction and spontaneous lactamization of **13C** was carried with trichlorosilane and diisopropylethylamine (DIEA) in acetonitrile/dichloromethane, using a coil reactor under continuous flow conditions to give (*S*)-Rolipram in 83% yield and 94% e.e.

### 6.2 Enantioselective C-C bond-formation through 1,2-addition in continuous flow system

**FIGURE 14:**
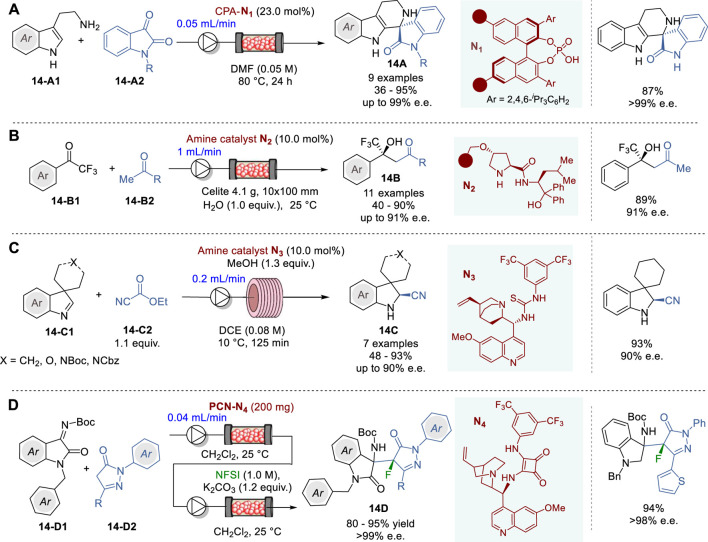
Enantioselective 1,2-addition reactions in continuous flow system **(A−D)**.

One of the most widely used methods for the synthesis of chiral tetrahydro-β-carbolines is the asymmetric Pictet−Spengler reaction. Thus, over the years many enantioselective Pictet-Spengler reactions of tryptamines with aldehydes using chiral organo/metal catalysts have been reported, however limited to only batch operations (Wanner et al., 2007; Stöckigt et al., 2011). In 2023, Pericàs and coworkers reported the first enantioselective Pictet-Spengler reaction of tryptamines **14-A1** and isatins **14-A2** under continuous flow conditions, using a polymer-supported CPA (*R*)-TRIP **N**_**1**_ in a packed bed reactor, to give the corresponding quarternary tetrahydro-β-carbolines (THβC) **14A** in 36–95% yield and 39–99% e.e. (Figure 14A) (Chaudhari et al., 2023). This continuous flow system was successuly applied to the synthesis of the chiral precursors of Tadalafil, the Iboga-type alkaloid (+)-Tabertinggine, and antimalarial spiroindolinones.

The use of chiral secondary amines as catalysts in asymmetric aldol reactions has been developed as a powerful method for the synthesis of optically active secondary or tertiary alcohols through asymmetric carbon-carbon bond formation. Thus, a few catalytic processes have been developed for the enantioselective synthesis of tertiary trifluoromethyl carbinols, but there are certain issues, such as long reaction time, high catalyst loading and racemization of the product, which needs to be addressed for practical use (Trost and Brindle, 2010; Pluta et al., 2019). In 2021, Kobayashi and coworkers reported the first catalytic enantioselective aldol reactions of trifluoroacetophenones **14-B1** with methyl ketones **14-B2** under continuous flow conditions, using a polystyrene-supported prolinamide catalyst **N**_**2**_ in a column reactor to give trifluoromethyl carbinols **14B** in 40–90% yield and 83–91% e.e. (Figure 14B) (Yue et al., 2021). It was found that a proper feeding of water facilitated the hydrolysis of the iminium intermediates on the polymer, which enabled unusually long life times (>195 h) of prolinamide catalyst **N**_**2**_ in this continuous flow system. A mechanistic study revealed that the continuous flow system suppressed the racemization of the product **14B** observed under batch conditions. The salient feature of this continuous flow process was demonstrated by its application to the formal synthesis of a chiral fenpentadiol analog.

In 2023, Brindisi and coworkers reported the first continuous flow asymmetric Strecker synthesis of spiroindolenines **14C** through enantioselective cyanation of cyclic (*Z*)-aldimines **14-C1** promoted by cinchona alkaloid-based organocatalyst using ethyl cyanoformate as the cyanide source (Figure 14C) (Alfano et al., 2023). Under optimized continuous flow conditions with the quinine-derived catalyst **N**_**3**_ using 0.08 M solution of reactants at a flow rate of 0.2 mL/min, several spiroindolenines **14C** were obtained in 48–93% yield and 50–90% e.e. Regarding sustainability, the reaction time was drastically reduced from 72 h under batch conditions to 125 min under continuous flow conditions, showing a far greater space-time-yield (STY) (0.24 g L^-1^h^-1^) in the flow system than that in batch system (0.0208 g L^-1^h^-1^).

In 2018, Zhao and coworkers reported the first successful use of porous carbon nanosheet (PCN) as a support for immobilizing chiral quinine-squaramide **N**_**4**_, and its application to the continuous flow synthesis of fluoropyrazolonylaminooxyindoles **14D** through asymmetric Friedel–Crafts addition of pyrazolones **14-D2** to isatin ketimines **14-D1** with *N*-fluoro-benzene-sulfonimide (NSFI) (Figure 14D) (Zhao et al., 2018). The 3-substitued-3-aminooxindoles motifs constitute the core structure of numerous natural products and drug candidates (Kaur et al., 2015). The PCN-quinine-squaramide catalyst **PCN-N**_**4**_ was prepared by immobilizing quinine-squaramide **N**_**4**_ on the vinylated PCN surface through free-radical co-polymerization of **N**_**4**_, bearing a vinyl group, using divinylbenzene (DVB) as a linker. For the surface modification, PCN was first reacted with diazonium salt generated *in situ* from 4-aminobenzyl alcohol and sodium nitrate to give 4-hydroxymethylphenyl-PCN. The subsequent acylation with acryloyl chloride afforded the vinyl-functionalized PCN intermediate (PCN-vinyl), which was ready for co-polymerization with **N**_**4**_ and DVB to give polymer-coated **PCN-N**_**4**_. The continuous flow reaction of **14-D1** with **14-D2** was performed, using a packed-bed reactor with **PCN-N**_**4**_ and the product solution was directly delivered to a column reactor with K_2_CO_3_ with NFSI feeding to give 14D in 80–90% yield and >99% e.e. (5 examples). Throughout the continuous flow operation, no decline in the catalytic efficiency of **PCN-N**_**4**_ was observed, which indicates that **N**_**4**_ attached to PCN is fully exposed to the reaction medium, resulting in a homogeneous catalytic environment for the active sites.

### 6.3 Asymmetric hydrogenation and carbon-heteroatom bond forming reactions in continuous flow system

**FIGURE 15:**
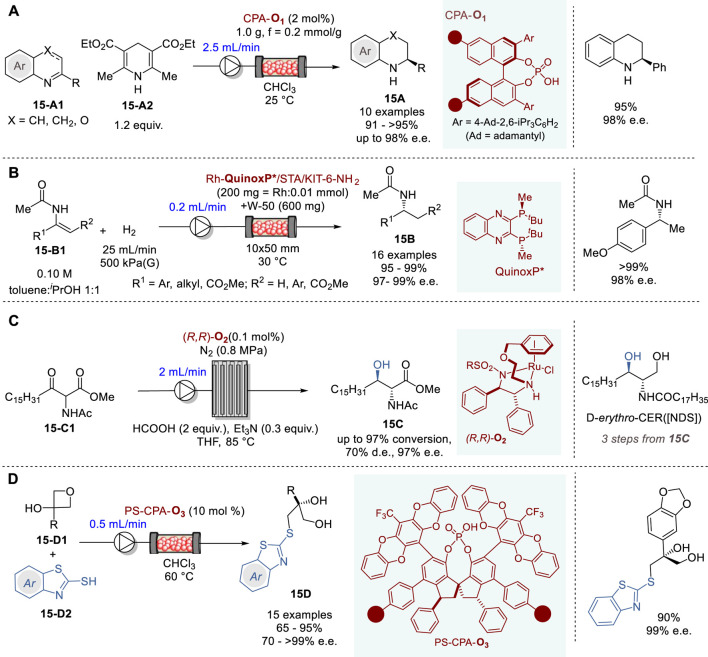
Asymmetric hydrogenation and carbon-heteroatom bond forming reactions in continuous flow system **(A−D)**.

Recently, transfer hydrogenation for the synthesis of chiral nitrogen-containing heterocycles has been attracting considerable attention (Parmar et al., 2014; Pálvölgyi et al., 2021; Faisca Phillips and Pombeiro, 2023). In 2023, Nagorny and coworkers reported an efficient transfer hydrogenation of 2-substituted quinolines (X = CH or CH_2_) and 3-substituted 1,4-benzooxazines (X = O) (**15-A1**) with a Hantzsch ester **15-A2** promoted by an immobilized chiral phosphoric acid catalyst CPA-**O**_**1**_ in a fluidized bed reactor under continuous flow conditions to give the corresponding tetrahydroquinolines (X = CH_2_) and dihydrobenzooxazines (X = O) **15A** in 91∼>95% yield and 86–98% e. e. (Figure 15A) (Zhelavskyi and Jhang, 2023). The immobilized CPA-**O**_**1**_ catalyst can be recovered and recycled by filtration and washing. In this reactor, the flow rate of 2.0–2.5 mL/min was optimal, yielding the product **15A** at 1.5 g (7.5 mmol) per hour.

In 2020, Kobayashi and coworkers reported a highly efficient continuous flow asymmetric hydrogenation of enamides and dehydroamino esters **15-B1** promoted by the immobilized chiral Rh-QuinoxP* catalyst on KIT-6 mesoporous silica to give the corresponding amides, α- and β-amino acid esters **15B** in excellent yield and enantiopurity (Figure 15B) (Saito and Kobayashi, 2020). Preparation of the chiral heterogenous Rh catalyst involved three steps, i.e., (i) the surface amine functionalization of KIT-6 with 3-aminopropyl-Si(OEt)_3_ coupling reagent to form KIT-6-NH_2_, (ii) salt formation with silicotungstic acid (STA) to form STA/KIT-6-NH_2_, and (iii) immobilization of chiral Rh complex Rh-QuinoxP*, which was prepared *in situ* from [Rh (nbd)_2_]^+^BF_4_^−^ and QinoxP* to give Rh-QuinoxP*/STA/KIT-6-NH_2_ catalyst. Then, a fixed-bed reactor was packed with the chiral heterogeneous catalyst and used for the continuous flow reaction under hydrogen gas flow. Various enamides, α- and β-dehydroamino esters were converted to the corresponding amides, α- and β-dehydroamino acid esters **15B** in 95∼>99% yield and 97–99% e.e. The chiral catalyst activity did not change for 90 h with a turnover number (TON) of 10,800.

The optically active ceramide *N*-((2*S*,3*R*)-1,3-dihydroxyoctadecan-2-yl)stearamide (D-erythro-CER [NDS]) is clinically used for the treatment of prominent skin diseases such as psoriasis and atopic dermatitis. In 2019, Touge and coworkers reported an efficient continuous flow asymmetric transfer hydrogenation process through dynamic kinetic resolution of methyl 2-acetamido-3-oxooctadecanoate (**15-C1**) catalyzed by a chiral diamine-Ru complex (*R,R*)-**O**_**2**_ to produce methyl (2*R*,3*R*)-2-acetamido-3-hydroxyoctadecanoate (**15C**) (Figure 15C) (Touge et al., 2018). The continuous flow reaction was executed by introducing **15-C1**, HCO_2_H, NEt_3_, and Ru–diamine catalyst (*R,R*)-**O**_**2**_ in THF into a pipes-in-series reactor. The reaction, conducted in a 100 L reactor, comprising 19 stainless steel pipes connected to 18 smaller diameter jumper tubes with 0.1 mol% catalyst (*R,R*)-**O**_**2**_ for 36 h, produced 77.4 kg of **15C** in 96% yield with 97% e. e. and 69% d. e. This product **15C** was converted to ceramide D-erythro-CER [NDS] in three steps, i.e., NaBH_4_ reduction, deacetylation with NaOH and amidation with methyl stearate, followed by recrystallization, wherein 58 kg of D-erythro-CER [NDS] with >99% d. e. and >99% e.e.

In 2020, Pericàs and coworkers reported the enantioselective desymmetrization of 3-substituted 3-hydroxyoxetanes **15-D1** with benzothiazole-2-thiols **15-D2** promoted by immobilized CPAs with very bulky 3,3′-diphenyl-SPINOLs (e.g., CPA-**O**_**3**_) to give the corresponding 3-benzothiazol-2-ylthiopropane-1,2-diol (**15D**) in high yield and enantiopurity (Figure 15D) (Lai et al., 2020). C2-symmetrical 1,1-spirobiindane-7,7-diol (SPINOL) derivatives containing polymerizable styryl units were prepared and subjected to radical co-polymerization with styrene to form the corresponding polystyrene anchored SPINOLs, which were reacted with POCl_3_/pyridine to give polymer anchored PS-CPAs. Six chiral PS-CPAs were screened for optimization and PS-CPA-**O**_**4**_ was identified as the best catalyst, giving a product **15D** in 90% yield and 98% e. e. in the model reaction for optimization. The excellent results achieved by PS-CPA-**O**_**3**_ could be attributed to the very bulky pentacyclic heteroaryl substituents at the 6 and 6′positions and their rotational mobility which plays a crucial role in achieving excellent enantioselectivity. The continuous flow reactions were carried out in a size-adjustable jacketed tubular reactor to give various 3-benzothiazol-2-ylthiopropane-1,2-diols (**15D**) in 65–95% yield and 70∼>99% e. e.

## 7 Conclusion and future perspective

Development of catalytic asymmetric reactions continues to be an important research topic in organic chemistry, partly because it will contribute significantly to the pharmaceutical sciences and production of therapeutic drugs. The ideal catalytic reactions would proceed in 100% yield with complete chemoselectivity, regioselectivity, and stereoselectivity. From the standpoint of green chemistry, highly efficient (i.e., high turnover number) and safe reagents are desirable. We have highlighted the most significant works on the catalytic asymmetric reactions from 2018 to 2023 in this review article, which clearly shows that significant advances have been made in this field in the last 5 years. In addition to metal catalysis and biocatalysis, organocatalysis has also proven to be a valuable tool for the construction of optically active compounds as evidenced by the Nobel Prize in Chemistry awarded jointly to Benjamin List and David MacMillan in 2021. Recently, electrocatalysis and photoredox catalysis have emerged as powerful tools for the development of new and valuable transformations for construction of organic molecules in enantiomerically pure form.

In addition to single catalyst systems, dual catalyst systems have significantly contributed to the development of novel enantioselective catalytic reactions. For example, hybrid catalyst systems, consisting of a transition metal catalyst and an organocatalyst are quite effective and efficient. Cooperative catalyst systems such as photoredox catalyst with transition metal catalyst, biocatalyst with electrocatalyst, and organocatalyst with photoredox catalyst, have also flourished. Furthermore, continuous flow system has made rapid advancement and will potentially lead the industrial production of chiral pharmaceutical drugs in the near future. Continuous flow system has an advantage over classical batch system such as ease of precise control of reaction conditions, and ease of scale-up for production. The future of catalytic asymmetric synthesis is highly promising, because of the continuous emergence of novel catalytic systems, while multi-catalyst systems will further expand their horizon. We hope this review article will help readers update and appreciate the remarkable recent progress in catalytic asymmetric synthesis and its bright future.
